# The avoidance of G-CSF and the addition of prophylactic corticosteroids after autologous stem cell transplantation for multiple myeloma patients appeal for the at-home setting to reduce readmission for neutropenic fever

**DOI:** 10.1371/journal.pone.0241778

**Published:** 2020-11-04

**Authors:** Luis-Gerardo Rodríguez-Lobato, Alexandra Martínez-Roca, Sandra Castaño-Díez, Alicia Palomino-Mosquera, Gonzalo Gutiérrez-García, Alexandra Pedraza, María Suárez-Lledó, Montserrat Rovira, Carmen Martínez, Carlos Fernández de Larrea, María-Teresa Cibeira, Laura Rosiñol, Ester Lozano, Pedro Marín, Joan Cid, Miquel Lozano, Ana Belén Moreno-Castaño, Marta Palomo, Maribel Díaz-Ricart, Cristina Gallego, Adelina Hernando, Susana Segura, Enric Carreras, Álvaro Urbano-Ispizua, Joan Bladé, Francesc Fernández-Avilés

**Affiliations:** 1 Department of Hematology, Home Care and Bone Marrow Transplantation Unit, Hospital Clínic of Barcelona, Barcelona, Spain; 2 Department of Hematology, Amyloidosis and Multiple Myeloma Unit, Hospital Clínic of Barcelona, Barcelona, Spain; 3 Institut d’Investigacions Biomèdiques August Pi i Sunyer (IDIBAPS), Barcelona, Spain; 4 Department of Hemotherapy and Hemostasis, Apheresis and Cellular Therapy Unit, Hospital Clínic of Barcelona, Barcelona, Spain; 5 Department of Hematopathology, Laboratory of Hemostasis and Eritropathology, Biomedical Diagnosis Center (CDB), Hospital Clínic of Barcelona, Barcelona, Spain; 6 Barcelona Endothelium Team (BET), Josep Carreras Leukemia Research Institute, Barcelona, Spain; 7 Josep Carreras Leukemia Research Institute, Hospital Clínic/University of Barcelona Campus, Barcelona, Spain; University of Kentucky, UNITED STATES

## Abstract

**Background:**

Autologous stem cell transplantation (ASCT) remains the standard of care for young multiple myeloma (MM) patients; indeed, at-home ASCT has been positioned as an appropriate therapeutic strategy. However, despite the use of prophylactic antibiotics, neutropenic fever (NF) and hospital readmissions continue to pose as the most important limitations in the outpatient setting. It is possible that the febrile episodes may have a non-infectious etiology, and engraftment syndrome could play a more significant role. The aim of this study was to analyze the impact of both G-CSF withdrawal and the addition of primary prophylaxis with corticosteroids after ASCT.

**Methods:**

Between January 2002 and August 2018, 111 MM patients conditioned with melphalan were managed at-home beginning +1 day after ASCT. Three groups were established: Group A (n = 33) received standard G-CSF post-ASCT; group B (n = 32) avoided G-CSF post-ASCT; group C (n = 46) avoided G-CSF yet added corticosteroid prophylaxis post-ASCT.

**Results:**

The incidence of NF among the groups was reduced (64%, 44%, and 24%; *P*<0.001), with a non-significant decrease in hospital readmissions as well (12%, 6%, and 2%; *P* = 0.07). The most important variables identified for NF were: HCT-CI >2 (OR 6.1; *P* = 0.002) and G-CSF avoidance plus corticosteroids (OR 0.1; *P*<0.001); and for hospital readmission: age ≥60 years (OR 14.6; *P* = 0.04) and G-CSF avoidance plus corticosteroids (OR 0.07; *P* = 0.05).

**Conclusions:**

G-CSF avoidance and corticosteroid prophylaxis post ASCT minimize the incidence of NF in MM patients undergoing at-home ASCT. This approach should be explored in a prospective randomized clinical trial.

## Introduction

The multiple myeloma (MM) treatment landscape has evolved dramatically over the last few years with the emergence of a next-generation proteasome inhibitor (carfilzomib), an immunomodulatory drug (pomalidomide) and monoclonal antibodies (daratumumab and elotuzumab) [1]. However, most guidelines continue to support high-dose therapy followed by autologous stem cell transplantation (ASCT) as the standard of care for newly diagnosed patients without severe comorbidities [2–5]. This treatment is considered a safe procedure with an extremely low transplant-related mortality (TRM) (<3%), due to improvements that include the use of peripheral blood-derived hematopoietic stem cell products and post-transplantation administration of granulocyte colony-stimulating factor (G-CSF) [6,7].

In recent years though, due to extensive waiting lists and an ever-growing concern about the proper use of health care resources, many groups have implemented outpatient ASCT programs. Such an approach has resulted in considerably acceptable outcomes in terms of hematopoietic recovery, toxicity and TRM; as well as improvements in cost and resource use, risk of infections, length of hospital admission and quality of life [8–12]. Nevertheless, neutropenic fever (NF) continues to pose as the most important limitation in the outpatient setting with an estimated incidence of 80–100%, which in most cases acts as a main driver for hospital readmissions [8,13–15]. In light of such an observation, the use of antibiotic prophylaxis during chemotherapy-induced neutropenia is recommended [16,17]. Since 2002, our group has enhanced infectious prophylaxis in the outpatient setting by adding ceftriaxone to levofloxacin, reducing the incidence of NF to 76% with a bacteremia rate of 26% and 8% in hospital readmissions [8]. It is possible that the high incidence of NF in spite of antibiotic prophylaxis could be a consequence of non-infectious causes [18]. Recently, the usefulness of G-CSF has been questioned; indeed, some transplant groups have stopped using it, observing no changes in relevant transplant outcomes and avoiding potential adverse effects including fatigue, bone pain, fever, engraftment syndrome (ES), and capillary leak syndrome [19,20]. In January 2011, our group too decided to follow suit and stop the routine use of G-CSF.

Engraftment syndrome is a syndromic entity occurring in the peri-engraftment period within the context of ASCT. It is characterized by non-infectious fever, skin rash, and diarrhea, resulting in less frequent hepatic dysfunction, transient encephalopathy, and capillary leak syndrome [21–23]. While symptoms are usually mild and transient, some patients may, however, develop complications, delay hospital discharge, require intensive care treatment or experience death [24]. The exact pathogenesis is not well understood; it is probable that the syndrome may arise in response to a release of pro-inflammatory cytokines (IL-2, TNF-α, IFN-γ, IL-8 and IL-6), associated with endothelial damage (high levels of vWF, sVCAM-1, sICAM-1, sTNFRI and low levels of ADAMTS-13 activity) and high levels of C-reactive protein [23–29]. The estimated incidence of ES ranged between 5% and 72% [30,31] depending on the diagnostic criteria implemented [21,22]. The onset of ES has been associated with ASCT for solid tumors (breast cancer), autoimmune diseases (multiple sclerosis) and monoclonal gammopathies (MM, AL amyloidosis and POEMS syndrome) that included the use of post-ASCT G-CSF, a high infused CD34^+^ cell dose, earlier and more rapid granulocyte recovery and the introduction of bortezomib and immunomodulatory drugs as induction therapy in MM [18,23–26,32–34]. The most important aspect in managing ES is early recognition, ruling out alternative causes, and the use of corticosteroids (methylprednisolone 1 to 1.5 mg/Kg/day for two or three days) following a quick tapering regimen [26]. Due to the dramatic response to corticosteroids, some authors have encouraged the pre-emptive or prophylactic use of such drugs [21,31,35]. In observance of such aspect, our ASCT team established primary corticosteroid prophylaxis after ASCT in January 2014.

The aim of this study was to analyze the outcomes of different prophylactic policies to reduce NF, ES and hospital readmissions in our at-home ASCT program for patients with MM in order to increase the safety of those treatments by which these patients undergo. First: the standard G-CSF administration after ASCT; second: the withdrawal of G-CSF after ASCT; and third: the addition of primary prophylaxis for ES with corticosteroids after ASCT.

## Materials and methods

### Patients

The clinical records of 853 consecutive ASCT for hematological malignancies at Hospital Clínic of Barcelona between January 2002 and August 2018 were reviewed. Patients who received ASCT in the in-patient setting were excluded (n = 539). Two hundred and three patients were excluded for being non-MM patients. The final study population was comprised of 111 MM patients (S1 Fig). Some of the patients were included in a previous publication (18); however, the series has increased and only outpatients have been included in the analysis. The study was approved by the Ethics Committee of the Hospital Clínic of Barcelona, and was in accordance with the Declaration of Helsinki. The eligibility criteria for at-home transplantation included ECOG≤2; travel time from home to the hospital <60 minutes; permanent caregiver availability. All patients signed an informed consent form.

### At-home ASCT program

Our at-home ASCT program is based on the early-discharge outpatient model and has been published elsewhere [8,36]. Data on prior induction treatment and peripheral stem cell mobilization regimen was collected. All patients received conditioning regimen with intravenous melphalan 200 mg/m^2^; they were discharged from hospital the day after stem cell infusion. Hematology nurses visited patients once a day and performed laboratory tests three times a week. Prevention of chemotherapy-induced nausea and vomiting, and prophylaxis for oral mucositis (OM) followed standard international supportive care protocols. Proton pump inhibitors or H2 antagonists were used in all patients. Red blood cell and platelet transfusion were administered when hemoglobin concentration was <8 g/dL and platelet count was ≤10 x 10^9^/L, respectively. All patients received antimicrobial prophylaxis with fluoroquinolone, fluconazole, acyclovir (if herpes serology was positive), aerosolized pentamidine and enhanced antimicrobial prophylaxis with ceftriaxone [8] (1 g/day) from day +1 until the appearance of fever or neutrophil engraftment. At the onset of the first episode of NF, clinical evaluation, collection of blood and urine cultures and X-ray/CT scan were performed, along with empirical antimicrobial therapy with meropenem (1 g IV t.i.d.). Treatment with teicoplanin was added in cases when WHO grade ≥ 2 mucositis and signs of central venous access (CVA) infection existed. Empirical antimicrobial therapy was maintained until patients were afebrile for at least three days with no signs of infection and an absolute neutrophil count (ANC) of ≥1x10^9^/L. When ES was suspected, methylprednisolone 1 mg/Kg/12h was administered for 3 days and then tapered over 7–8 days. Indications for hospital readmission were WHO grade 4 mucositis, uncontrolled vomiting or diarrhea, hemodynamic instability, respiratory distress, and willingness of caregiver or patient. At-home program discharge criteria consisted of an ANC of ≥1x10^9^/L and afebrile status without antibiotic administration for a minimum of 48 hours.

### Prophylactic strategies to reduce neutropenic fever

Patients were divided into three groups in the outpatient setting during three different time periods established in our transplant unit per the NF. Group A (January 2002 to December 2010) included all patients who received standard G-CSF (filgrastim) 5 μg/Kg/day beginning on post-transplant day +7 until ANC reached 1x10^9^/L for three consecutive days; group B (January 2011 to December 2013) included patients who did not receive G-CSF post-transplant; and group C (January 2014 to August 2018) included patients who avoided G-CSF post-transplant, but did undergo primary prophylaxis with corticosteroids for ES with oral prednisone 0.5 mg/Kg/day from day+7 until their ANC reached 0.5x10^9^/L for three consecutive days.

### Definitions

Neutrophil and platelet engraftment were defined as the first of three consecutive days, in which an ANC >0.5x10^9^/L and platelet count >20x10^9^/L without platelet transfusion were achieved, respectively. NF was defined as a newly observed temperature ≥38°C with an ANC of <0.5x10^9^/L and bacteremia as isolation of bacteria from a blood culture with fever or symptoms and/or signs of infection. ES was defined using Maiolino clinical criteria [21]. Non-infectious fever was defined as a new fever without clinical or microbiological documentation or response to antimicrobials. Skin rash was defined as macular-papular exanthema mimicking acute graft *versus* host disease involving >25% of body surface area. Pulmonary infiltrates were considered secondary to ES in the absence of clinical and laboratory evidence of infection, cardiac failure, or pulmonary embolism. Diarrhea was defined as ≥2 episodes of liquid stool per day without microbiologically documented infection. Weight gain was defined >2.5% increase from baseline body weight. Hepatic and renal dysfunction was defined by either an elevation in serum bilirubin ≥2mg/dL or an increase in serum AST or ALT of ≥2 times the upper limit of normal (ULN) and serum creatinine of ≥2 times the ULN. Transient encephalopathy was defined as confusion not secondary to any other etiology.

### Statistical analysis

Descriptive statistical analysis was performed. Median and range were used for continuous variables; meanwhile, frequency and percentage were used for categorical variables. Patient variables and outcomes were compared using Fisher’s exact test or χ^2^ test for categorical variables, as well as *t*-test or Wilcoxon rank-sum test for continuous variables. Univariate and multivariate binary logistic regression models were used to identify factors associated with NF, ES, and hospital readmission. Logistic regression analysis was performed by a backward stepwise method. The cumulative incidence of NF, ES, and hospital readmission were calculated with Gray’s test, as well as the Fine and Gray proportional hazard model for the analysis of the sub-distribution of competitive risks [37,38]. The probability of progression-free survival (PFS) and overall survival (OS) was calculated with the Kaplan-Meier estimator; survival curves were compared with the log-rank test. The starting point for time-to-event analysis was the date of ASCT [39]. All *p* values were two-sided with statistical significance evaluated at the 0.05 alpha level. All analyses were performed using R.3.6.1 (R Foundation for Statistical Computing, Vienna, Austria, http://www.R-project.org).

## Results

### Patient characteristics

Patient demographics and clinical characteristics are summarized in Table 1. The median age was 56 years (range: 25–70) and 70 patients (63%) were males. As expected, the most frequent isotypes of heavy and light chain produced were IgG (49%) and Kappa (62%), respectively. Most patients had an International Staging System (ISS) of I (63%). Bortezomib-based schemes were received as an induction regimen in 84% of patients and 26% received ≥2 treatment lines before ASCT. The underlying disease was in complete response in 36% of the patients; meanwhile, 64% were in very good partial response or worse. Twenty-four patients (22%) had a hematopoietic cell transplantation-comorbidity index (HCT-CI) score of >2. The median infused CD34^+^ cell dose was 3.3 x 10^6^/Kg (range, 1.7–9.4). Thirty-three patients (30%) received G-CSF post-ASCT (group A); 32 patients (29%) did not receive G-CSF post-ASCT (group B), and 46 patients (41%) avoided G-CSF post-ASCT and received primary prophylaxis with corticosteroids (group C). The groups were similar in most basal characteristics. However, we observed more ISS grade III patients in group C in comparison to group A (A: 6% *vs*. C: 19.5%; *P* = 0.003) and a greater number of patients in group B and C received bortezomib-based schemes (A: 58%, B: 91% and C: 98%; *P*<0.001). The median time of prednisone prophylaxis in group C was 8 days (range 1–14). While in the subgroup of patients who did not develop and did develop ES, the median time of prednisone prophylaxis were 9 days (range 5–14) and 2 days (range 1–4), respectively. Thirty-nine patients (39%) developed ES; their distributions according to symptoms were: fever (100%), diarrhea (86%), skin rash (28%), and pulmonary infiltrates (7%). The median follow-up of all patients was 39.8 months.

**Table 1:** Main patient characteristics.

Characteristics	Total group (n = 111)	Group A (n = 33)	Group B (n = 32)	Group C (n = 46)	*P*		
					A *vs*. B	B *vs*. C	A *vs*. C
Age (range)	56 (25–70)	51 (25–67)	57 (40–69)	58 (39–70)	**0.03**	0.71	0.06
Gender, male (%)	70 (63)	20 (61)	18 (56)	32 (69.6)	0.72	0.23	0.41
Immunological subtype					0.89	0.36	0.85
IgG (%)	54 (49)	17 (52)	18 (56)	19 (41.3)			
IgA (%)	29 (26)	8 (24)	9 (28)	12 (26.1)			
Bence Jones (%)	25 (23)	7 (21)	5 (16)	13 (28.4)			
Light chain isotype, Kappa (%)	69 (62)	19 (58)	17 (53)	33 (71.7)	0.71	0.14	0.23
ISS					0.55	**0.04**	**0.003**
I (%)	70 (63)	27 (82)	23 (72)	20 (43.5)			
II (%)	28 (25)	4 (12)	7 (22)	17 (37.09)			
III (%)	13 (12)	2 (6)	2 (6)	9 (19.5)			
Number of pre-ASCT lines ≥ 2 (%)	29 (26)	8 (24)	8 (25)	13 (28.3)	1.00	0.8	0.8
Pre-transplant therapy					**0.002**	0.16	**<0.001**
Chemotherapy (%)	18 (16)	14 (42)	3 (9)	1 (2.2)			
Bortezomib-based schemes (%)	93 (84)	19 (58)	29 (91)	45 (97.8)			
Response before ASCT					0.23	0.53	0.64
CR (%)	40 (36)	15 (46)	8 (25)	17 (37.0)			
VGPR/PR (%)	65 (59)	16 (49)	22 (69)	27 (58.7)			
MR/progression (%)	6 (5)	2 (5)	2 (6)	2 (4.3)			
Mobilization					0.20	0.10	0.08
G-CSF (%)	92 (83)	31 (94)	26 (82)	35 (76)			
G-CSF + cyclophosphamide (%)	5 (5)	2 (6)	3 (9)	0 (0)			
Plerixafor (%)	14 (12)	0 (0)	3 (9)				
HCT-CI >2 (%)	24 (22)	4 (12)	8 (25)	12 (26.1)	0.18	0.91	0.13
CD34^+^ x10^6^/Kg (range)	3.3 (1.7–9.4)	3.2 (1.9–6.4)	3.0 (1.9–9.4)	3.6 (1.7–7.2)	0.57	0.51	0.14

Group A: G-CSF without corticosteroids; Group B: No G-CSF without corticosteroids; Group C: No G-CSF adding corticosteroids.

ASCT: autologous stem cell transplantation; CR: complete response; HCT-CI: hematopoietic cell transplantation-comorbidity index; ISS: international staging system; MR: minimal response; PR: partial response; VGPR: very good partial response.

### Patient outcomes

**Fig 1:**
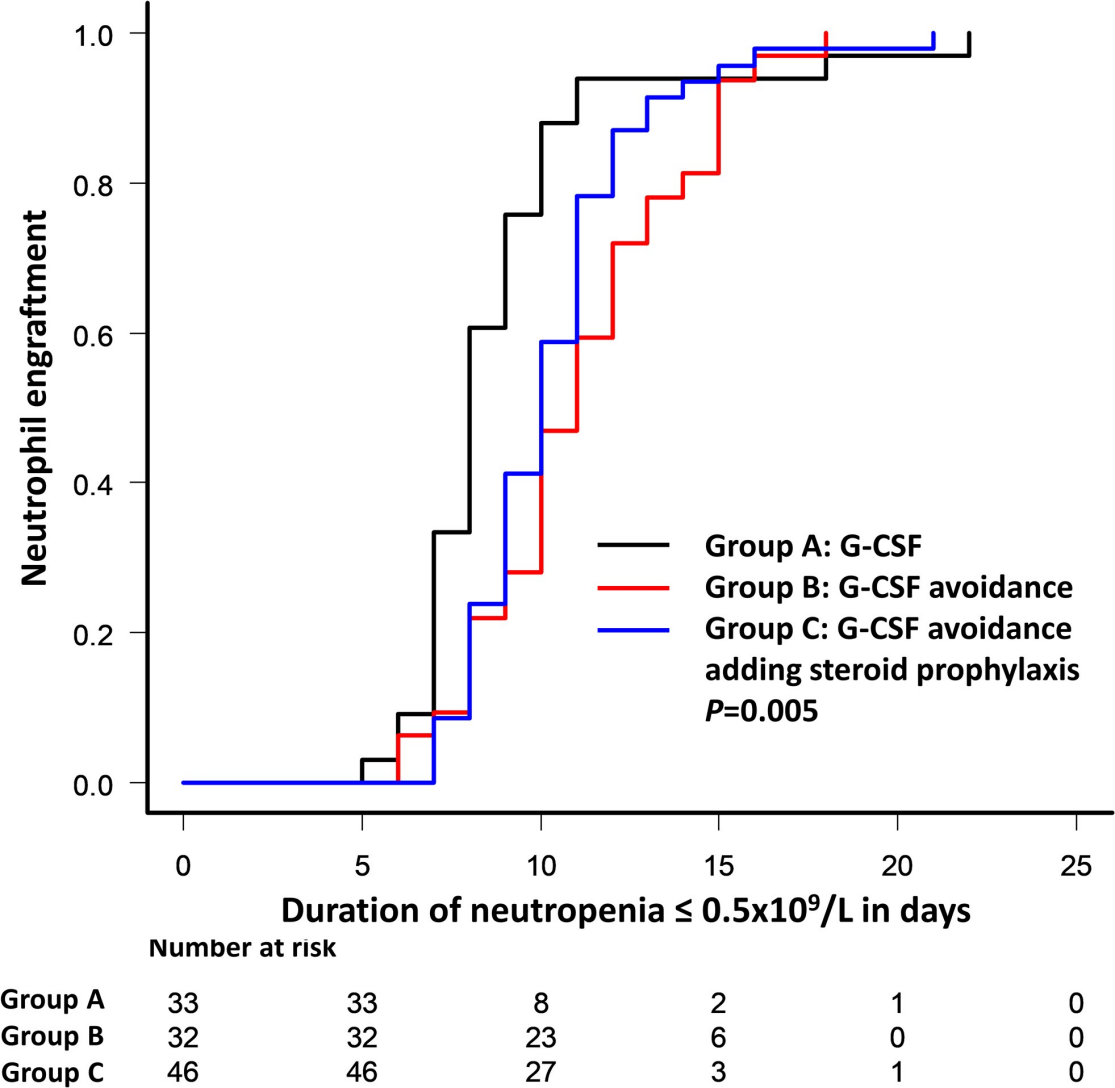
Cumulative incidence of neutrophil engraftment comparing group A (G-CSF without corticosteroid), group B (avoiding G-CSF and corticosteroid), group C (avoiding G-CSF and adding corticosteroid) during the first 30 days after autologous stem cell transplantation.

The most important clinical outcomes are summarized in Table 2. There was a significant difference in duration of severe neutropenia between patients that received G-CSF and those who did not, with a median of 8, 11, and 10 days in group A, B and C, respectively (A *vs*. B; *P* = 0.005 and A *vs*. C; *P* = 0.04). The 10-day cumulative incidence of neutrophil engraftment was 88% (95% confidence interval (CI), 70–95%) in group A, 47% (95%CI, 26–62%) in group B, and 59% (95%CI, 42–71%) in group C (*P* = 0.005) (Fig 1). There were no differences among groups in either platelet engraftment or their requirement for platelet transfusions.

**Table 2:** Clinical outcomes.

Characteristics	Group A (n = 33)	Group B (n = 32)	Group C (n = 46)	*P*		
				A *vs*. B	B *vs*. C	A *vs*. C
**Engraftment**						
First day of neutropenia ≤ 0.5x10^9^/L (range)	4 (0–5)	4 (1–5)	4 (3–6)	0.31	0.07	**0.009**
Duration of neutropenia ≤ 0.5x10^9^/L (range)	8 (5–22)	11 (6–18)	10 (7–21)	**0.005**	0.24	**0.04**
Duration of thrombocytopenia ≤ 20,000x10^9^/L (range)	12 (0–37)	12 (9–17)	11 (0–34)	0.37	0.58	0.27
**Fever**						
Neutropenic fever (≥38°C) (%)	21 (64)	14 (44)	11 (24)	0.11	0.07	**0.0004**
First day with fever (range)	7.5 (4–12)	8 (3–11)	8 (5–9)	0.47	0.34	0.75
Duration of fever (range)	2 (1–5)	1 (1–5)	3 (1–5)	0.09	**0.05**	0.56
Positive blood cultures (%)	2/21 (10)	2/14 (14)	1/11 (2)	1.00	1.00	1.00
**Engraftment syndrome**	19 (58)	14 (44)	10 (22)	0.27	**0.04**	**0.001**
Fever (%)	19 (100)	14 (100)	10 (100)			
Rash (%)	5 (26)	4 (29)	3 (30)			
Diarrhea (%)	15 (79)	12 (86)	10 (100)			
Pulmonary infiltrates (%)	3 (16)	0 (0)	0 (0)			
**Antibiotic use**						
Days with antibiotic prophylaxis, median (range)	8 (5–11)	11 (6–16)	10 (7–21)	**<0.001**	0.44	**<0.001**
Without further presence of fever	4 (0–9)	4 (0–8)	4 (0–6)	0.18	0.23	0.79
Further presence of fever	0.3	0.2	0.09	**<0.001**	**<0.001**	**<0.001**
Days with antibiotic treatment	0.09	0.08	0.02	0.56	**<0.001**	**<0.001**
Meropenem daily dose/outpatient-days*	0.04	0	0.004	**<0.001**	0.27	**<0.001**
Teicoplanin daily dose/outpatient-days*						
Amikacin daily dose/outpatient-days*						
**Toxicity**						
Mucositis ≥ 2 (%)	2 (6)	0 (0)	2 (4)	0.49	0.51	1.00
Cutaneous ≥ 2 (%)	0 (0)	0 (0)	0 (0)	1.00	1.00	1.00
Diarrhea ≥ 2 (%)	1 (3)	0 (0)	9 (20)	1.00	1.00	0.08
Nausea and vomiting ≥ 2 (%)	0 (0)	0 (0)	1 (2)	1.00	1.00	1.00
**Hospital readmission**						
Readmissions (%)						
Duration of readmission	4 (12)	2 (6)	1 (2)	0.35	0.66	0.07
(range)	8 (4–13)	2.5 (2–39)	12	0.06	-	-

Group A: G-CSF without corticosteroids; Group B: No G-CSF without corticosteroids; Group C: No G-CSF adding corticosteroids.

* Total sum of antibiotic days in the sample/total sum of days of sample follow-up.

### Neutropenic fever

**Fig 2:**
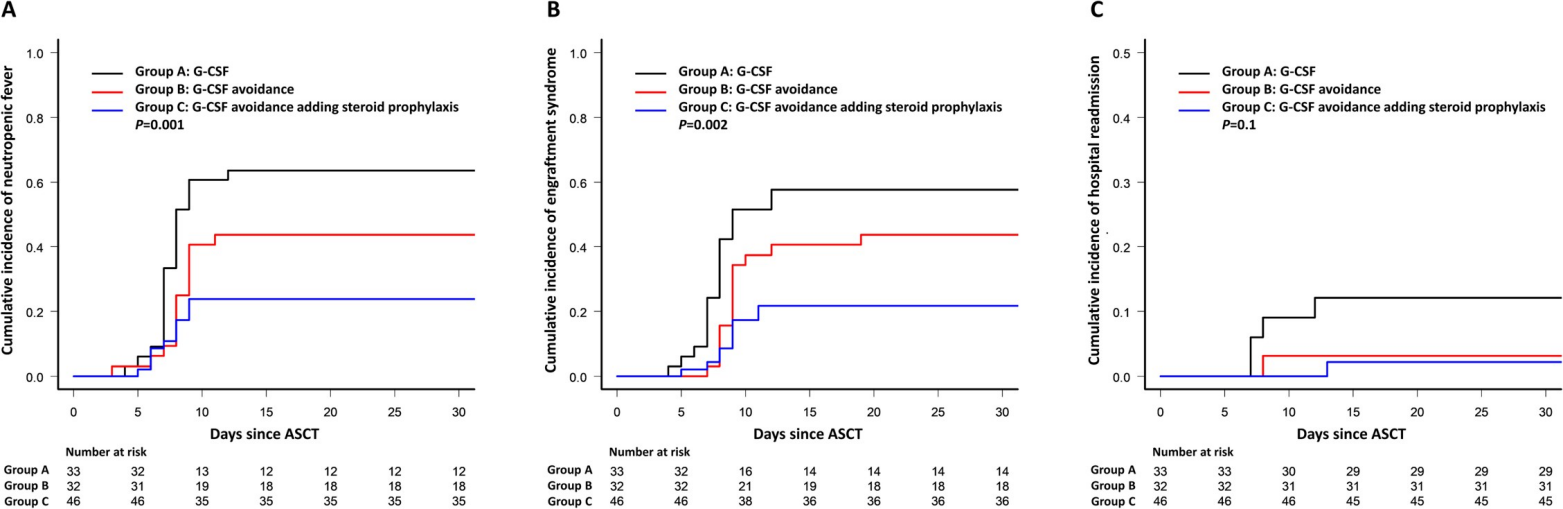
A. Cumulative incidence of neutropenic fever comparing group A (G-CSF without corticosteroid), group B (avoiding G-CSF and corticosteroid), group C (avoiding G-CSF and adding corticosteroid) during the first 30 days after autologous stem cell transplantation. B. Cumulative incidence of engraftment syndrome comparing group A (G-CSF without corticosteroid), group B (avoiding G-CSF and corticosteroid), group C (avoiding G-CSF and adding corticosteroid) during the first 30 days after autologous stem cell transplantation. C. Cumulative incidence of hospital readmission comparing group A (G-CSF without corticosteroid), group B (avoiding G-CSF and corticosteroid), group C (avoiding G-CSF and adding corticosteroid) during the first 30 days after autologous stem cell transplantation.

There was a significant reduction in NF incidence between group A and group C (64% *vs*. 24%; *P*<0.001), with a relative risk reduction of 0.38 (95%CI, 0.21–0.67; *P*<0.001) and a number needed to treat of 2.52 (95%CI, 1.7–5.1). Onset and duration of NF were similar among groups. In the multivariate binary logistic regression analysis for NF, HCT-CI >2 was a risk factor (odds ratio (OR) 6.1; *P* = 0.002), while the avoidance of G-CSF and the addition of corticosteroids (group C) were protective (OR 0.1; *P*<0.001) (Table 3). The 10-day cumulative incidence of NF were 61% in group A, 41% in group B, and 24% in group C (*P* = 0.001) (Fig 2A). In the competing risk regression model for NF, the avoidance of G-CSF and the addition of corticosteroids retained their independent protective factor (hazard ratio (HR) 0.53; *P*<0.001) and HCT-CI >2 as risk factor (HR 2.24; *P*<0.01). Regarding bacteremia, there were no differences in the number of positive blood cultures in all three groups (A: 2 isolations; B: 2 isolations; C: 1 isolation), and all were positive for coagulase-negative *Staphylococcus*. During follow-up, there was a case of *Clostridioides difficile*-associated diarrhea in each two groups (A and C), which responded to oral vancomycin treatment.

**Table 3:** Multivariate regression model for neutropenic fever, engraftment syndrome, and hospital readmission.

Characteristic	OR	95% CI	*P*
**Neutropenic fever**			
Gender, female	1.8	0.7–4.6	0.2
HCT-CI > 2	6.1	1.9–19.4	**0.002**
Novel drugs*	0.7	0.2–2.6	0.6
No G-CSF with corticosteroid	0.1	0.02–0.4	**0.0007**
**Engraftment syndrome**			
Gender, female	2.3	0.9–5.6	0.07
HCT-CI > 2	4.0	1.4–11.4	**0.01**
Novel drugs*	0.4	0.1–1.3	0.1
No G-CSF with corticosteroid	0.2	0.1–0.8	**0.02**
**Hospital readmission**			
Age ≥ 60 years	14.6	1.1–19.9	**0.04**
No G-CSF with corticosteroid	0.07	0.01–0.99	**0.05**

* Proteasome inhibitors (bortezomib) and immunomodulatory drugs (thalidomide and lenalidomide).

Regarding the ES, its incidence decreased with the non-administration of G-CSF and the addition of prophylactic corticosteroids (group A: 58% *vs*, group C: 22%; *P* = 0.001). The 10-day cumulative incidence of ES was 52% in group A, 38% in group B, and 17% in group C (*P* = 0.002) (Fig 2B). In the multivariate analysis, the most important variables related to the development of ES were female gender (OR 2.3; *P* = 0.05), HCT-CI >2 (OR 4.0; *P* = 0.01), and group C (OR 0.2; *P* = 0.02) (Table 3). The use of corticosteroids in group C did not increase the incidence of viral and fungal infections.

### Antibiotic use

Patients who received G-CSF (Group A) and did not develop NF received fewer days of prophylactic antibiotic compared with Group B and C (A: 8 days; B: 11 days; C: 10 days; A *vs*. B or C; *P*<0.001) related to the duration of neutropenia. Respecting antibiotic treatment in patients who presented NF, Groups A and B received more days of antibiotic compared with group C (expressed in daily dose/outpatient-days) (meropenem: 0.3 *vs*. 0.2 *vs*. 0.09; *P*<0.001; teicoplanin: 0.09 *vs*. 0.08 *vs*. 0.02; *P*<0.001; amikacin: 0.04 *vs*. 0 *vs*. 0.004; *P*<0.001; Table 2).

### Toxicity and hospital readmission

The non-administration of G-CSF with the addition of prophylactic corticosteroids did not modify the incidence and grade of mucositis, or other subtypes of toxicities including cutaneous, diarrhea, nausea, and vomiting (Table 2). Hospital readmission was necessary for four patients (12%) within group A, two patients (6%) within group B, and one patient (2%) within group C (A *vs*. C; *P* = 0.07). The causes of readmissions were ES (n = 3) and respiratory syncytial virus (RSV) pneumonia (n = 1) in group A; ES (n = 1) and RSV pneumonia in group B; and human metapneumovirus infection (n = 1) in group C. In the multivariate analysis for hospital readmission, age ≥ 60 years was a risk factor (OR 14.6; *P* = 0.04); meanwhile, the avoidance of G-CSF with the administration of corticosteroid retained an independent protective factor (OR 0.07; *P* = 0.05) (Table 3). Regarding adverse events related to prednisone prophylaxis in group C, no cases of suppression of the hypothalamic-pituitary-adrenal axis were found, nor was it observed toxicity ≥2 at the metabolic and cardiovascular levels. In the one-year follow-up, we did not observe an increase in the incidence of viral and fungal infections in those patients who received prophylactic prednisone.

### Transplant outcomes

**Fig 3:**
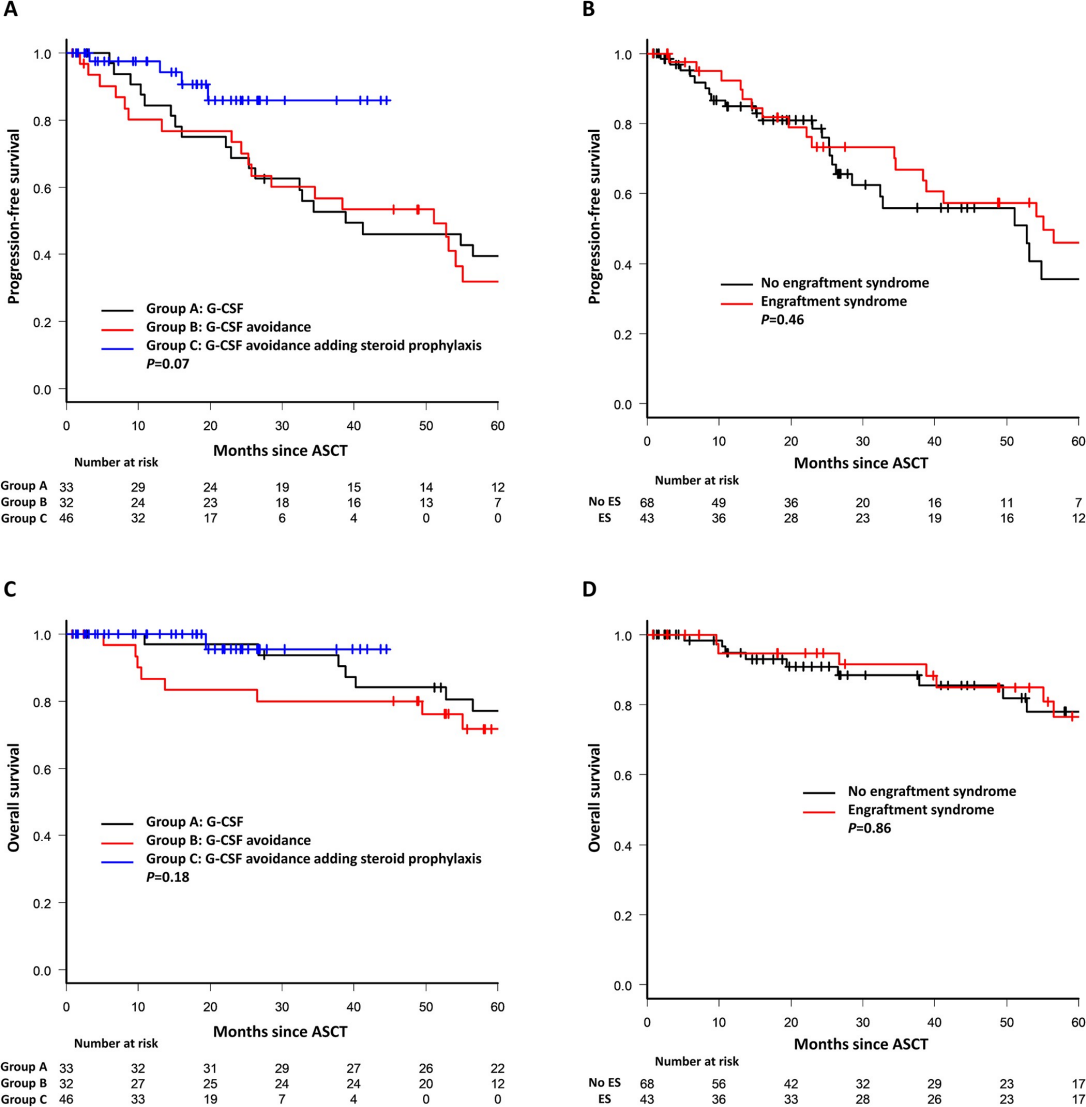
A. Progression-free survival comparing group A, group B, and group C; B. Progression-free survival in all patients with and without engraftment syndrome; C. Overall survival comparing group A, group B, and group C; D. Overall survival in all patients with and without engraftment syndrome.

The one-year TRM in the whole series was 0.9% with no statistically significant differences among groups. The only death recorded occurred in group B on day +35 due to RSV pneumonia. There were no differences in PFS or OS between patients with and without NF, ES, and the avoidance of G-CSF with the addition of corticosteroids. However, there was a substantial and expected improvement in group C PFS probably due to the use of novel drugs in the induction regimen (Fig 3A–3D).

## Discussion

This study shows that patients with MM who received high-dose chemotherapy with melphalan and peripheral ASCT in the outpatient setting observe an increase in safely managing and reducing the incidence of NF. Such changes to support that conclusion include enhanced antibiotic prophylaxis with levofloxacin plus ceftriaxone; avoidance of G-CSF; and the addition of primary prophylaxis with corticosteroids after transplantation. The withdrawal of G-CSF and the use of corticosteroids did not increase the rate of infections nor did they modify transplant outcomes.

NF remains one of the most important concerns associated with ASCT in the outpatient setting. Despite the use of peripheral blood-derive hematopoietic stem cells and enhanced infectious prophylaxis, the incidence of NF and hospital readmissions in patients with MM remains high (30–80% and 8–33%, respectively) [11,14,15,40,41]. Our group has incorporated the combination of levofloxacin plus ceftriaxone into the prophylactic antibiotic armamentarium, reducing the incidence of NF and hospital readmission to 76% and 8%, respectively [8]. Trying to bolster at-home strategies to reduce these complications and assuming that febrile episodes may have a non-infectious etiology, we decided to withdraw the use of G-CSF and add corticosteroids after ASCT. Studies published 25 years ago that validate the use of G-CSF cannot be taken into account to support its use today; the value of growth factors must be reevaluated with current supportive care [19]. In this study, the duration of severe neutropenia was longer in those groups who did not receive G-CSF post-ASCT, with a difference of two days. However, avoiding G-CSF did not modify the platelet engraftment, transfusion support, number of infections, or chemotherapy-related toxicity, which is consistent with other publications where the use of G-CSF is rationalized [18–20]. The introduction of the two preventive measures (group C) led to a 90% decrease in odds for NF onset, entailing that 2.5 patients would need to avoid G-CSF and receive corticosteroids in order to reduce one episode of NF and make it a useful strategy in the outpatient setting. Risk factors associated with NF are diverse; nonetheless, in many series there is a strong correlation with older age, advanced stage disease, infusion of <5x10^6^ CD34^+^/Kg, CVA infections, and OM [42]. In this regard, the multivariate analysis revealed an HCT-CI >2 as risk factor, hinting that patients with greater number and severity of comorbidities are at greater risk of developing fever.

The incidence of ES in the group A of patients was 58% while in group C, it was 22%. Our new policy of G-CSF avoidance post-transplant and the use of primary prophylaxis with corticosteroids have remarkably reduced ES incidence. These results are consistent with those published by Mossad *et al*., in which ES was reduced from 57% to 6% [31]. Although the physiopathology of ES is not fully understood, it is possible that some pro-inflammatory risk factors for developing ES, such as older age, HCT-CI >2 and pre-ASCT treatment ≥2 lines could be associated with the development of this complication [18]. In contrast to other studies, we did not observe any association among the amount of infused CD34^+^ cell dose, mobilization regimen, and use of novel anti-MM drugs in the development of ES.

The avoidance of G-CSF and the administration of corticosteroids did not boost the incidence of viral and fungal infections; it is likely due to the small variation in the number of days of severe neutropenia among groups and due to short use of corticosteroids. Regarding bacterial infections, the most frequently isolated bacteria was coagulase-negative *Staphylococcus* in all three groups, with these results being similar to those of other publications [12,43]. Regarding *Clostridioides difficile*-associated diarrhea, we observed two cases, which agree with the 0 and 13% incidence rates published by other groups [8,12,14,15,43].

In terms of toxicity, the non-administration of G-CSF and the addition of corticosteroid did not modify the incidence and grade of OM, ratifying the importance of better oral health care and cryotherapy [44]. The observed hospital readmission rate was quite low in all groups. We were only able to observe a trend to reduce it in group C. It is difficult to carry out adequate comparisons with other published series, since the incidence of hospital readmission is very variable (8–87%), dependent on the model of outpatient transplant program, ECOG, comorbidities, subtype of hematological disease and proficiency of the at-home unit, among other elements [8,10–12,15].

This study has some limitations as a retrospective, non-randomized, single-center-based study. The study spans a long period and we could not assess the continuous improvement in supportive care and enhanced proficiency of physicians and nurses who care for these patients. However, patients in group C were older, with worse ISS, and had the same infused CD34^+^ cell dose compared with group A. Notwithstanding, we observed a decrease in the incidence rate of NF and in hospital readmission, with no noted increase in adverse effects. We could not perform economic analysis; however, currently the median cost per day in our at-home ASCT program is €117, while the median hospital cost per day is €862, which represents important savings resulted mainly from lower hospitalization charges. Another benefit of our at-home transplant program is the ability to decrease the number of ward patients receiving an ASCT, and thus implement complex procedures, including haploidentical hematopoietic cell transplants and chimeric antigen receptor T-cells without increasing the number of hospital beds [45]. Finally, the outpatient setting could become a potential approach to maintaining hematopoietic transplants during the COVID-19 pandemic.

In conclusion, this study suggests that for patients with multiple myeloma in at-home ASCT, the avoidance of G-CSF and the addition of primary prophylaxis with corticosteroids after ASCT minimize the incidence rates of NF and ES. This preventive policy might not compromise transplant outcomes or increase the infection rate, and could provide clinicians with greater safety in the outpatient management of these patients. This approach should be explored in a prospective randomized multicenter study to confirm the results. Major confounding factors not accounted for in this small study may have led to the results.

## Supporting information

S1 Fig Flowchart of patient inclusion. (PDF)

S1 Database (PDF)
