# Supplementary figures and images for: The avoidance of G-CSF and the addition of prophylactic corticosteroids after autologous stem cell transplantation for multiple myeloma patients appeal for the at-home setting to reduce readmission for neutropenic fever
Source: PLoS One. 2020 Nov 4;15(11):e0241778. doi: 10.1371/journal.pone.0241778 (PMC7641449; doi:10.1371/journal.pone.0241778)

Supporting information:

Supplementary Figure 1. Flowchart of patient inclusion.

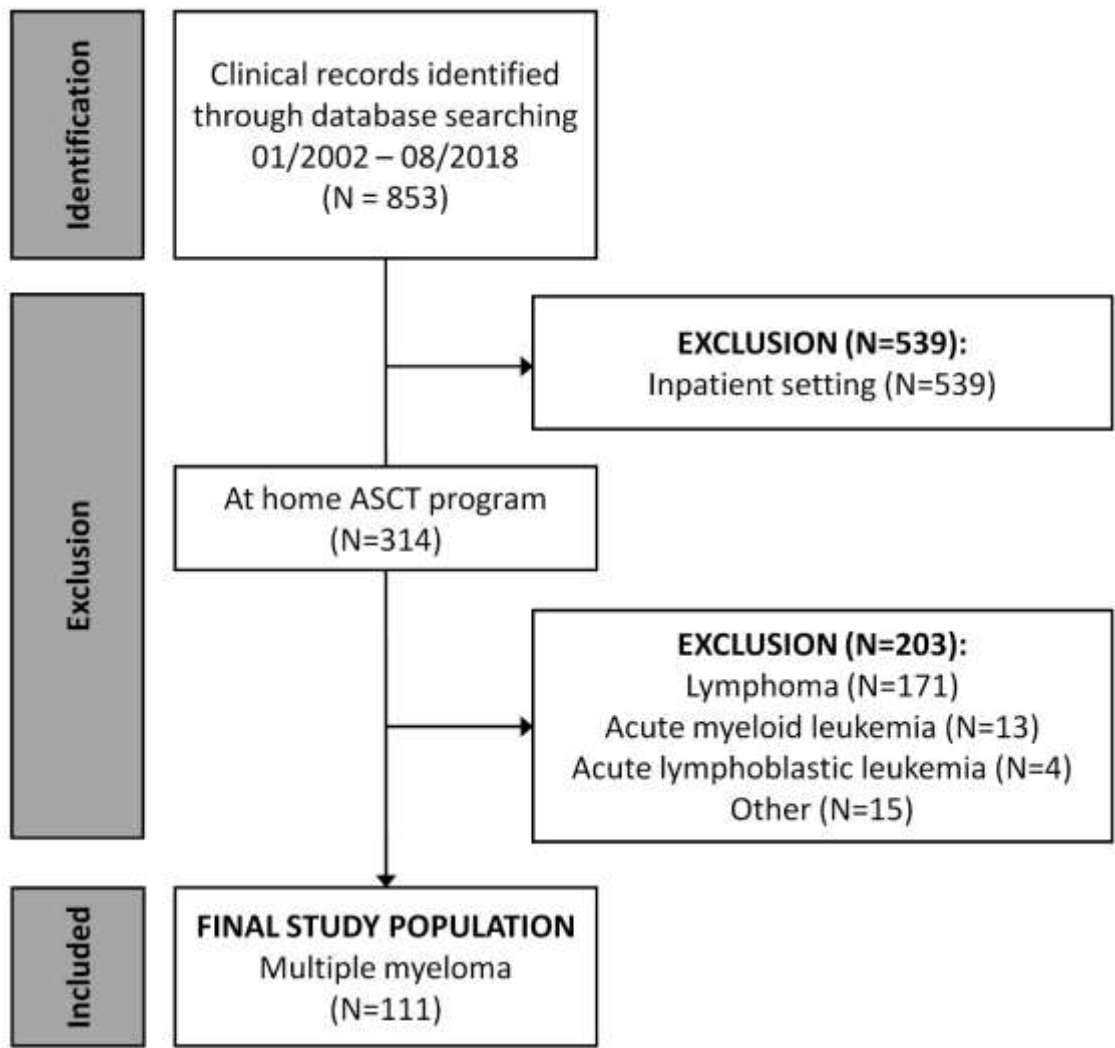

Supplement: S1 Fig — (PDF) [file pone.0241778.s001.pdf]
