# Supplementary material for: The avoidance of G-CSF and the addition of prophylactic corticosteroids after autologous stem cell transplantation for multiple myeloma patients appeal for the at-home setting to reduce readmission for neutropenic fever
Source: PLoS One. 2020 Nov 4;15(11):e0241778. doi: 10.1371/journal.pone.0241778 (PMC7641449; doi:10.1371/journal.pone.0241778)
Supplement: S1 Database — (PDF) [file pone.0241778.s002.pdf]

| Number | Age | Sex | ASCTdate | CD34+ | Neutr>500 | CortProfDays | elet_engraftm | Plat>20000 | Day 500 | Day < 500 |
|---|---|---|---|---|---|---|---|---|---|---|
| 1 | 43 | 1V | 27/02/2001 | 5,8 | 13 | 0 | 1 | 16 | 4 | 03/03/2001 |
| 2 | 46 | 1V | 16/05/2002 | 3,6 | 13 | 0 | 1 | 13 | 4 | 20/05/2002 |
| 3 | 58 | 1V | 28/05/2002 | 2,2 | 11 | 0 | 1 | 13 | 5 | 02/06/2002 |
| 4 | 57 | 2M | 26/07/2002 | 3,2 | 11 | 0 | 1 | 12 | 0 | 26/07/2002 |
| 5 | 64 | 1V | 25/06/2002 | 2,6 | 12 | 0 | 1 | 13 | 4 | 29/06/2002 |
| 6 | 42 | 1V | 03/02/2003 | 5,7 | 11 | 0 | 1 | 11 | 3 | 06/02/2003 |
| 7 | 41 | 1V | 10/06/2003 | 3,8 | 15 | 0 | 1 | 13 | 5 | 15/06/2003 |
| 8 | 51 | 2M | 10/10/2003 | 2 | 12 | 0 | 1 | 10 | 4 | 14/10/2003 |
| 9 | 44 | 1V | 23/02/2004 | 2,5 | 11 | 0 | 1 | 10 | 5 | 28/02/2004 |
| 10 | 25 | 1V | 14/02/2005 | 2,5 | 12 | 0 | 1 | 11 | 3 | 17/02/2005 |
| 11 | 44 | 1V | 07/03/2005 | 2,1 | 10 | 0 | 1 | 10 | 0 | 07/03/2005 |
| 12 | 40 | 2M | 25/04/2006 | 3,4 | 11 | 0 | 1 | 10 | 3 | 28/04/2006 |
| 13 | 55 | 2M | 22/05/2006 | 3,5 | 26 | 0 | 1 | 0 | 4 | 26/05/2006 |
| 14 | 41 | 2M | 19/09/2006 | 4,4 | 22 | 0 | 1 | 37 | 3 | 22/09/2006 |
| 15 | 62 | 2M | 16/04/2007 | 2,6 | 12 | 0 | 1 | 21 | 3 | 19/04/2007 |
| 16 | 48 | 1V | 15/10/2007 | 3,4 | 14 | 0 | 1 | 21 | 4 | 19/10/2007 |
| 17 | 64 | 1V | 12/11/2007 | 3,4 | 12 | 0 | 1 | 12 | 5 | 17/11/2007 |
| 18 | 34 | 1V | 23/11/2007 | 1,9 | 11 | 0 | 1 | 10 | 4 | 27/11/2007 |
| 19 | 47 | 1V | 12/09/2008 | 3,8 | 12 | 0 | 1 | 13 | 3 | 15/09/2008 |
| 20 | 64 | 1V | 10/03/2009 | 2,6 | 11 | 0 | 1 | 13 | 3 | 13/03/2009 |
| 21 | 64 | 1V | 09/12/2009 | 3,3 | 12 | 0 | 1 | 10 | 4 | 13/12/2009 |
| 22 | 48 | 1V | 22/03/2010 | 4,4 | 11 | 0 | 1 | 11 | 4 | 26/03/2010 |
| 23 | 61 | 1V | 02/07/2010 | 2,4 | 11 | 0 | 1 | 15 | 4 | 06/07/2010 |
| 24 | 52 | 1V | 02/09/2010 | 2,1 | 12 | 0 | 1 | 15 | 3 | 05/09/2010 |
| 25 | 48 | 2M | 18/02/2011 | 3,2 | 11 | 0 | 1 | 13 | 4 | 22/02/2011 |
| 26 | 58 | 2M | 15/03/2011 | 6,4 | 11 | 0 | 1 | 11 | 4 | 19/03/2011 |
| 27 | 41 | 2M | 04/04/2011 | 5,4 | 10 | 0 | 1 | 10 | 3 | 07/04/2011 |
| 28 | 62 | 2M | 16/05/2011 | 3,1 | 10 | 0 | 1 | 12 | 3 | 19/05/2011 |
| 29 | 56 | 1V | 03/06/2011 | 4,8 | 16 | 0 | 1 | 20 | 5 | 08/06/2011 |
| 30 | 64 | 2M | 01/07/2011 | 2,5 | 11 | 0 | 1 | 11 | 3 | 04/07/2011 |
| 31 | 65 | 2M | 12/08/2011 | 2,6 | 11 | 0 | 1 | 12 | 3 | 15/08/2011 |
| 32 | 67 | 1V | 05/09/2011 | 2,1 | 12 | 0 | 1 | 13 | 3 | 08/09/2011 |
| 33 | 61 | 2M | 03/11/2011 | 2,1 | 15 | 0 | 1 | 13 | 4 | 07/11/2011 |
| 34 | 49 | 2M | 05/12/2011 | 2,4 | 15 | 0 | 1 | 11 | 3 | 08/12/2011 |
| 35 | 59 | 2M | 16/03/2012 | 3,2 | 14 | 0 | 1 | 11 | 4 | 20/03/2012 |
| 36 | 53 | 2M | 02/04/2012 | 3 | 12 | 0 | 1 | 12 | 3 | 05/04/2012 |
| 37 | 52 | 1V | 16/04/2012 | 2,8 | 15 | 0 | 1 | 11 | 3 | 19/04/2012 |
| 38 | 59 | 1V | 04/06/2012 | 3,6 | 18 | 0 | 1 | 13 | 4 | 08/06/2012 |
| 39 | 65 | 1V | 16/07/2012 | 3,9 | 19 | 0 | 1 | 15 | 4 | 20/07/2012 |

| | | | | | | | | | | |
|---|---|---|---|---|---|---|---|---|---|---|
| 40 | 57 | 1V | 31/07/2012 | 5,1 | 18 | 0 | 1 | 16 | 3 | 03/08/2012 |
| 41 | 62 | 2M | 13/09/2012 | 4 | 17 | 0 | 1 | 11 | 3 | 16/09/2012 |
| 42 | 61 | 2M | 12/11/2012 | 2,6 | 14 | 0 | 1 | 12 | 3 | 15/11/2012 |
| 43 | 56 | 1V | 04/12/2012 | 2,3 | 18 | 0 | 1 | 13 | 3 | 07/12/2012 |
| 44 | 47 | 1V | 21/12/2012 | 2,9 | 13 | 0 | 1 | 12 | 5 | 26/12/2012 |
| 45 | 61 | 2M | 31/12/2012 | 2,7 | 15 | 0 | 1 | 12 | 4 | 04/01/2013 |
| 46 | 52 | 1V | 14/01/2013 | 2,5 | 14 | 0 | 1 | 14 | 1 | 15/01/2013 |
| 47 | 48 | 2M | 22/02/2013 | 5,93 | 11 | 0 | 1 | 11 | 5 | 27/02/2013 |
| 48 | 61 | 1V | 18/03/2013 | 2,1 | 16 | 0 | 1 | 12 | 4 | 22/03/2013 |
| 49 | 52 | 2M | 02/04/2013 | 2,7 | 20 | 0 | 1 | 11 | 4 | 06/04/2013 |
| 50 | 45 | 2M | 20/06/2013 | 2,4 | 13 | 0 | 1 | 13 | 3 | 23/06/2013 |
| 51 | 56 | 2M | 12/07/2013 | 2,6 | 13 | 0 | 1 | 13 | 5 | 17/07/2013 |
| 52 | 49 | 1V | 06/08/2013 | 2,5 | 17 | 0 | 1 | 13 | 3 | 09/08/2013 |
| 53 | 63 | 2M | 21/10/2013 | 2 | 20 | 0 | 1 | 17 | 3 | 24/10/2013 |
| 54 | 62 | 1V | 11/11/2013 | 4,2 | 15 | 0 | 1 | 11 | 3 | 14/11/2013 |
| 55 | 66 | 1V | 14/11/2013 | 3,4 | 14 | 0 | 1 | 11 | 4 | 18/11/2013 |
| 56 | 66 | 1V | 29/11/2013 | 4 | 11 | 0 | 1 | 11 | 5 | 04/12/2013 |
| 57 | 50 | 1V | 09/12/2013 | 9,4 | 14 | 0 | 1 | 9 | 5 | 14/12/2013 |
| 58 | 53 | 1V | 14/03/2014 | 5 | 13 | 0 | 1 | 12 | 5 | 19/03/2014 |
| 59 | 40 | 1V | 27/03/2014 | 5,7 | 12 | 0 | 1 | 11 | 4 | 31/03/2014 |
| 60 | 56 | 1V | 12/05/2014 | 3 | 16 | 0 | 1 | 14 | 5 | 17/05/2014 |
| 61 | 69 | 1V | 01/07/2014 | 3,5 | 13 | 0 | 1 | 12 | 3 | 04/07/2014 |
| 62 | 62 | 1V | 19/09/2014 | 2,2 | 14 | 0 | 1 | 12 | 4 | 23/09/2014 |
| 63 | 56 | 2M | 13/10/2014 | 5,9 | 12 | 0 | 1 | 10 | 3 | 16/10/2014 |
| 64 | 63 | 2M | 17/10/2014 | 1,9 | 15 | 0 | 1 | 11 | 4 | 21/10/2014 |
| 65 | 48 | 2M | 27/10/2014 | 3,6 | 13 | 0 | 1 | 11 | 4 | 31/10/2014 |
| 66 | 50 | 1V | 26/01/2015 | 4 | 16 | 10 | 1 | 12 | 5 | 31/01/2015 |
| 67 | 40 | 1V | 23/02/2015 | 5,2 | 12 | 5 | 1 | 11 | 5 | 28/02/2015 |
| 68 | 41 | 1V | 24/03/2015 | 2,7 | 16 | 9 | 1 | 10 | 3 | 27/03/2015 |
| 69 | 49 | 1V | 13/04/2015 | 2,9 | 14 | 8 | 1 | 10 | 3 | 16/04/2015 |
| 70 | 60 | 1V | 18/06/2015 | 3,3 | 12 | 2 | 1 | 10 | 4 | 22/06/2015 |
| 71 | 55 | 1V | 07/09/2015 | 2,5 | 11 | 7 | 1 | 11 | 4 | 11/09/2015 |
| 72 | 67 | 1V | 14/03/2016 | 4,4 | 11 | 7 | 1 | 10 | 3 | 17/03/2016 |
| 73 | 48 | 1V | 25/04/2016 | 2,3 | 13 | 7 | 1 | 12 | 3 | 28/04/2016 |
| 74 | 61 | 1V | 13/06/2016 | 5,9 | 13 | 7 | 1 | 12 | 4 | 17/06/2016 |
| 75 | 65 | 2M | 04/07/2016 | 3,8 | 12 | 8 | 1 | 0 | 4 | 08/07/2016 |
| 76 | 46 | 1V | 11/07/2016 | 3,4 | 16 | 9 | 1 | 13 | 3 | 14/07/2016 |
| 77 | 48 | 1V | 16/08/2016 | 2 | 12 | 7 | 1 | 13 | 4 | 20/08/2016 |
| 78 | 55 | 1V | 16/08/2016 | 5,3 | 13 | 7 | 1 | 15 | 4 | 23/08/2016 |
| 79 | 45 | 1V | 01/09/2016 | 3,7 | 15 | 9 | 1 | 15 | 4 | 05/09/2016 |

| | | | | | | | | | | |
|---|---|---|---|---|---|---|---|---|---|---|
| 80 | 45 | 2M | 15/09/2016 | 5,3 | 12 | 2 | 1 | 11 | 3 | 18/09/2016 |
| 81 | 49 | 2M | 07/10/2016 | 4,6 | 15 | 12 | 1 | 11 | 5 | 12/10/2016 |
| 82 | 64 | 2M | 24/10/2016 | 3,1 | 11 | 1 | 1 | 10 | 3 | 27/10/2016 |
| 83 | 64 | 1V | 28/10/2016 | 4,4 | 14 | 10 | 1 | 0 | 5 | 02/11/2016 |
| 84 | 63 | 1V | 22/11/2016 | 2 | 15 | 10 | 1 | 11 | 5 | 27/11/2016 |
| 85 | 64 | 2M | 05/12/2016 | 3,6 | 13 | 7 | 1 | 10 | 4 | 09/12/2016 |
| 86 | 56 | 1V | 12/12/2016 | 4,9 | 11 | 2 | 1 | 10 | 5 | 17/12/2016 |
| 87 | 51 | 1V | 19/01/2017 | 3,3 | 13 | 7 | 1 | 12 | 5 | 24/01/2017 |
| 88 | 62 | 1V | 06/02/2017 | 3 | 16 | 11 | 1 | 15 | 4 | 10/02/2017 |
| 89 | 58 | 2M | 10/02/2017 | 4,3 | 14 | 8 | 1 | 10 | 3 | 13/02/2017 |
| 90 | 50 | 1V | 03/03/2017 | 2,7 | 16 | 10 | 1 | 10 | 5 | 08/03/2017 |
| 91 | 63 | 1V | 09/03/2017 | 2 | 14 | 4 | 1 | 10 | 4 | 13/03/2017 |
| 92 | 62 | 1V | 13/03/2017 | 3 | 15 | 9 | 1 | 11 | 4 | 17/03/2017 |
| 93 | 45 | 2M | 30/03/2017 | 2,1 | 13 | 3 | 1 | 0 | 4 | 03/04/2017 |
| 94 | 57 | 1V | 31/03/2017 | 3,3 | 14 | 14 | 1 | 10 | 3 | 03/03/2017 |
| 95 | 39 | 2M | 05/04/2017 | 4,9 | 14 | 4 | 1 | 19 | 5 | 10/04/2017 |
| 96 | 61 | 1V | 02/05/2017 | 7,2 | 13 | 9 | 1 | 11 | 4 | 06/05/2017 |
| 97 | 45 | 2M | 08/05/2017 | 3,4 | 14 | 9 | 1 | 11 | 3 | 11/05/2017 |
| 98 | 64 | 1V | 13/07/2017 | 3,5 | 13 | 12 | 1 | 11 | 5 | 18/07/2017 |
| 99 | 62 | 2M | 20/07/2017 | 2,4 | 11 | 10 | 1 | 11 | 4 | 24/07/2017 |
| 100 | 62 | 1V | 07/08/2017 | 4,9 | 11 | 9 | 1 | 10 | 4 | 11/08/2017 |
| 101 | 58 | 1V | 11/08/2017 | 4,3 | 21 | 8 | 1 | 18 | 6 | 17/08/2017 |
| 102 | 63 | 2M | 29/09/2017 | 2,6 | 14 | 9 | 1 | 0 | 5 | 04/10/2017 |
| 103 | 40 | 1V | 11/12/2017 | 3,7 | 15 | 9 | 1 | 13 | 5 | 16/12/2017 |
| 104 | 63 | 2M | 01/03/2018 | 1,9 | 18 | 1 | 1 | 34 | 3 | 04/03/2018 |
| 105 | 59 | 1V | 04/04/2018 | 4,6 | 15 | 9 | 1 | 12 | 3 | 07/04/2018 |
| 106 | 70 | 1V | 04/05/2018 | 2,8 | 13 | 1 | 1 | 18 | 5 | 09/05/2018 |
| 107 | 65 | 1V | 04/06/2018 | 5,8 | 14 | 8 | 1 | 12 | 4 | 08/04/2018 |
| 108 | 56 | 1V | 29/06/2018 | 4,3 | 14 | 8 | 1 | 11 | 6 | 05/07/2018 |
| 109 | 58 | 2M | 23/07/2018 | 7,2 | **21** | 9 | 1 | 24 | 5 | 28/07/2018 |
| 110 | 69 | 2M | 07/08/2018 | 4,2 | 13 | 2 | 1 | 12 | 3 | 10/08/2018 |
| 111 | 55 | 1V | 13/08/2018 | 1,7 | 16 | 9 | 1 | 16 | 5 | 18/08/2018 |

| Days < 500 | Day 100 | Day < 100 | Days <100 | Fever>38ºC | Fever fist day | Fever_Event_days | ever_Duratio | Initial ATB | Days ATB1 |
|---|---|---|---|---|---|---|---|---|---|
| 9 | 5 | 04/03/2001 | 7 | 1 | 6 | 6 | 1 | 5 | 13 |
| 9 | 5 | 21/05/2002 | 6 | 0 | 0 | 35 | 0 | 0 | 0 |
| 6 | 6 | 03/06/2002 | 5 | 1 | 7 | 7 | 1 | 1 | 7 |
| 10 | 0 | 26/07/2002 | 10 | 1 | 9 | 9 | 2 | 1 | 5 |
| 8 | 5 | 30/06/2002 | 6 | 0 | 0 | 35 | 0 | 0 | 0 |
| 8 | 4 | 07/02/2003 | 6 | 0 | 0 | 35 | 0 | 0 | 0 |
| 9 | 5 | 15/06/2003 | 3 | 1 | 5 | 5 | 5 | 1 | 10 |
| 8 | 5 | 15/10/2003 | 6 | 0 | 0 | 35 | 0 | 0 | 0 |
| 5 | 6 | 01/03/2004 | 3 | 0 | 0 | 35 | 0 | 0 | 0 |
| 8 | 4 | 18/02/2005 | 6 | 0 | 0 | 35 | 0 | 0 | 0 |
| 11 | 7 | 14/03/2005 | 3 | 1 | 8 | 8 | 2 | 1 | 3 |
| 9 | 5 | 30/04/2006 | 6 | 0 | 0 | 35 | 0 | 0 | 0 |
| 22 | 5 | 27/05/2006 | 17 | 1 | 9 | 9 | 5 | 1 | 3 |
| 18 | 5 | 24/09/2006 | 7 | 1 | 12 | 12 | 2 | 1 | 12 |
| 10 | 5 | 21/04/2007 | 6 | 1 | 7 | 7 | 1 | 1 | 12 |
| 10 | 6 | 21/10/2007 | 6 | 0 | 0 | 35 | 0 | 0 | 0 |
| 7 | 6 | 18/11/2007 | 5 | 0 | 0 | 35 | 0 | 0 | 0 |
| 7 | 5 | 28/11/2007 | 5 | 1 | 8 | 8 | 2 | 1 | 6 |
| 8 | 4 | 16/09/2008 | 7 | 1 | 8 | 8 | 2 | 6 | 4 |
| 8 | 4 | 14/03/2009 | 7 | 1 | 7 | 7 | 2 | 0 | 0 |
| 8 | 6 | 15/12/2009 | 4 | 1 | 4 | 4 | 4 | 1 | 10 |
| 7 | 5 | 27/03/2010 | 4 | 1 | 7 | 7 | 4 | 1 | 8 |
| 7 | 5 | 07/07/2010 | 4 | 1 | 7 | 7 | 4 | 6 | 8 |
| 8 | 4 | 06/09/2010 | 6 | 1 | 8 | 8 | 3 | 1 | 7 |
| 7 | 5 | 28/02/2011 | 5 | 1 | 7 | 7 | 2 | 1 | 6 |
| 7 | 5 | 20/03/2011 | 5 | 1 | 7 | 7 | 1 | 1 | 7 |
| 7 | 5 | 09/04/2011 | 4 | 0 | 0 | 35 | 0 | 0 | 0 |
| 6 | 4 | 20/05/2011 | 4 | 1 | 7 | 7 | 2 | 1 | 4 |
| 10 | 6 | 09/06/2011 | 5 | 1 | 8 | 8 | 5 | 1 | 9 |
| 8 | 6 | 07/07/2011 | 3 | 0 | 0 | 35 | 0 | 0 | 0 |
| 7 | 5 | 17/08/2011 | 5 | 1 | 9 | 9 | 1 | 1 | 5 |
| 9 | 4 | 09/09/2011 | 7 | 1 | 8 | 8 | 1 | 1 | 7 |
| 12 | 5 | 08/11/2011 | 7 | 0 | 0 | 35 | 0 | 0 | 0 |
| 11 | 5 | 10/12/2011 | 9 | 0 | 0 | 35 | 0 | 0 | 0 |
| 10 | 5 | 21/03/2012 | 7 | 0 | 0 | 35 | 0 | 0 | 0 |
| 10 | 4 | 06/04/2012 | 7 | 0 | 0 | 35 | 0 | 0 | 0 |
| 10 | 4 | 20/04/2012 | 8 | 0 | 0 | 35 | 0 | 0 | 0 |
| 13 | 5 | 09/06/2012 | 7 | 1 | 9 | 9 | 1 | 1 | 8 |
| 15 | 5 | 21/07/2012 | 10 | 0 | 0 | 35 | 0 | 1 | 5 |

| | | | | | | | | | |
|---|---|---|---|---|---|---|---|---|---|
| 15 | 5 | 05/08/2012 | 8 | 1 | 11 | 11 | 2 | 1 | 7 |
| 14 | 7 | 20/09/2012 | 8 | 1 | 8 | 8 | 1 | 1 | 7 |
| 11 | 5 | 17/11/2012 | 6 | 1 | 7 | 7 | 1 | 1 | 8 |
| 15 | 4 | 08/12/2012 | 7 | 1 | 9 | 9 | 1 | 7 | 10 |
| 8 | 6 | 27/12/2012 | 6 | 0 | 0 | 35 | 0 | 0 | 0 |
| 11 | 6 | 06/01/2013 | 6 | 0 | 0 | 35 | 0 | 0 | 0 |
| 13 | 1 | 15/01/2013 | 12 | 0 | 0 | 35 | 0 | 0 | 0 |
| 6 | 5 | 27/02/2013 | 5 | 1 | 9 | 9 | 2 | 1 | 4 |
| 12 | 5 | 23/03/2013 | 9 | 0 | 0 | 35 | 0 | 0 | 0 |
| 16 | 5 | 07/04/2013 | 5 | 0 | 0 | 35 | 0 | 0 | 0 |
| 11 | 3 | 23/06/2013 | 9 | 1 | 3 | 3 | 1 | 1 | 7 |
| 8 | 6 | 18/07/2013 | 5 | 0 | 0 | 35 | 0 | 0 | 0 |
| 15 | 4 | 10/08/2013 | 7 | 0 | 0 | 35 | 0 | 0 | 0 |
| 18 | 4 | 25/10/2013 | 13 | 1 | 8 | 8 | 1 | 1 | 6 |
| 12 | 4 | 14/11/2013 | 9 | 0 | 0 | 35 | 0 | 0 | 0 |
| 10 | 5 | 19/11/2013 | 7 | 0 | 0 | 35 | 0 | 0 | 0 |
| 6 | 6 | 05/12/2013 | 4 | 0 | 0 | 35 | 0 | 0 | 0 |
| 7 | 5 | 14/12/2013 | 6 | 1 | 9 | 9 | 2 | 6 | 3 |
| 8 | 6 | 20/03/2014 | 6 | 1 | 6 | 6 | 5 | 1 | 4 |
| 8 | 5 | 01/04/2014 | 6 | 1 | 8 | 8 | 1 | 1 | 4 |
| 11 | 5 | 17/05/2014 | 7 | 0 | 0 | 35 | 0 | 0 | 0 |
| 10 | 4 | 05/07/2014 | 7 | 0 | 0 | 35 | 0 | 0 | 0 |
| 9 | 5 | 24/09/2014 | 6 | 1 | 8 | 8 | 3 | 6 | 7 |
| 9 | 4 | 17/10/2014 | 7 | 1 | 9 | 9 | 1 | 1 | 6 |
| 12 | 5 | 22/10/2014 | 9 | 0 | 0 | 35 | 0 | 0 | 0 |
| 10 | 5 | 01/11/2014 | 7 | 1 | 8 | 8 | 2 | 1 | 6 |
| 12 | 6 | 01/02/2015 | 9 | 0 | 0 | 35 | 0 | 0 | 0 |
| 7 | 6 | 01/03/2015 | 6 | 0 | 0 | 35 | 0 | 0 | 0 |
| 14 | 4 | 28/03/2015 | 11 | 0 | 0 | 35 | 0 | 0 | 0 |
| 11 | 5 | 18/04/2015 | 8 | 0 | 0 | 35 | 0 | 0 | 0 |
| 8 | 5 | 23/06/2015 | 6 | 1 | 6 | 6 | 5 | 1 | 7 |
| 7 | 5 | 12/09/2015 | 5 | 0 | 0 | 35 | 2 | 0 | 0 |
| 9 | 5 | 19/03/2016 | 6 | 0 | 0 | 35 | 0 | 0 | 0 |
| 10 | 4 | 29/04/2016 | 8 | 0 | 0 | 35 | 0 | 0 | 0 |
| 10 | 5 | 18/06/2016 | 6 | 0 | 0 | 35 | 0 | 0 | 0 |
| 10 | 5 | 09/07/2016 | 7 | 0 | 0 | 35 | 0 | 0 | 0 |
| 13 | 5 | 16/07/2016 | 9 | 0 | 0 | 35 | 0 | 0 | 0 |
| 9 | 5 | 21/08/2016 | 7 | 0 | 0 | 35 | 0 | 0 | 0 |
| 8 | 5 | 24/08/2016 | 7 | 0 | 0 | 35 | 0 | 0 | 0 |
| 10 | 5 | 06/09/2016 | 7 | 0 | 0 | 35 | 0 | 0 | 0 |

| | | | | | | | | | |
|---|---|---|---|---|---|---|---|---|---|
| 9 | 4 | 19/09/2016 | 7 | 1 | 9 | 9 | 1 | 1 | 5 |
| 10 | 6 | 13/10/2016 | 7 | 0 | 0 | 35 | 0 | 0 | 0 |
| 9 | 4 | 28/10/2016 | 7 | 1 | 8 | 8 | 3 | 1 | 6 |
| 10 | 6 | 03/11/2016 | 7 | 0 | 0 | 35 | 0 | 0 | 0 |
| 11 | 6 | 28/11/2016 | 7 | 0 | 0 | 35 | 0 | 0 | 0 |
| 9 | 5 | 10/12/2016 | 7 | 1 | 7 | 7 | 2 | 1 | 7 |
| 7 | 6 | 18/12/2016 | 4 | 1 | 6 | 6 | 4 | 6 | 5 |
| 8 | 6 | 25/01/2017 | 7 | 0 | 0 | 35 | 0 | 0 | 0 |
| 13 | 5 | 11/02/2017 | 9 | 0 | 0 | 35 | 0 | 0 | 0 |
| 11 | 5 | 15/02/2017 | 7 | 0 | 0 | 35 | 0 | 0 | 0 |
| 12 | 6 | 09/03/2017 | 8 | 0 | 0 | 35 | 0 | 0 | 0 |
| 10 | 5 | 14/03/2017 | 7 | 1 | 6 | 6 | 5 | 1 | 4 |
| 11 | 5 | 18/03/2017 | 7 | 0 | 0 | 35 | 0 | 0 | 0 |
| 9 | 5 | 04/04/2017 | 7 | 1 | 9 | 9 | 1 | 1 | 5 |
| 11 | 4 | 04/04/2017 | 9 | 0 | 0 | 35 | 0 | 0 | 0 |
| 9 | 6 | 11/04/2017 | 7 | 1 | 9 | 9 | 3 | 1 | 5 |
| 9 | 5 | 07/05/2017 | 7 | 0 | 0 | 35 | 0 | 0 | 0 |
| 11 | 4 | 12/05/2017 | 8 | 0 | 0 | 35 | 0 | 0 | 0 |
| 8 | 6 | 19/07/2017 | 6 | 0 | 0 | 35 | 0 | 0 | 0 |
| 7 | 5 | 25/07/2017 | 5 | 0 | 0 | 35 | 0 | 0 | 0 |
| 8 | 5 | 12/08/2017 | 5 | 0 | 0 | 35 | 0 | 0 | 0 |
| 15 | 7 | 18/08/2017 | 8 | 0 | 0 | 35 | 0 | 0 | 0 |
| 10 | 6 | 05/10/2017 | 6 | 0 | 0 | 35 | 0 | 0 | 0 |
| 11 | 6 | 17/12/2017 | 8 | 0 | 0 | 35 | 0 | 0 | 0 |
| 16 | 4 | 05/03/2018 | 9 | 1 | 8 | 8 | 3 | 1 | 5 |
| 12 | 5 | 09/04/2018 | 8 | 0 | 0 | 35 | 0 | 0 | 0 |
| 8 | 6 | 10/05/2013 | 5 | 1 | 5 | 5 | 2 | 6 | 7 |
| 11 | 5 | 09/04/2018 | 8 | 0 | 0 | 35 | 0 | 0 | 0 |
| 8 | 7 | 06/07/2018 | 6 | 0 | 0 | 35 | 0 | 0 | 0 |
| **21** | 6 | 29/07/2018 | 8 | 0 | 0 | 35 | 2 | 0 | 0 |
| 11 | 4 | 11/08/2018 | 7 | 1 | 8 | 8 | 2 | 6 | 6 |
| 12 | 6 | 19/08/2018 | 8 | 0 | 0 | 35 | 0 | 0 | 0 |

| ATB2 | ATB2 Which | Days ATB2 | ATB3 | ATB3 Which | Days ATB3 | Anfo-B | Blood Culture | Urine culture | G-CSF Days | Group |
|---|---|---|---|---|---|---|---|---|---|---|
| 1 | Amika | 4 | 1 | Teico | 4 | 0 | 0 | 0 | 7 | 0 |
| 0 | | | 0 | | | 0 | 0 | 0 | 8 | 0 |
| 0 | | | 0 | | | 0 | 0 | 0 | 7 | 0 |
| 0 | | | 0 | | | 0 | 0 | 0 | 6 | 0 |
| 0 | | | 0 | | | 0 | 0 | 0 | 6 | 0 |
| 0 | | | 0 | | | 0 | 0 | 0 | 5 | 0 |
| 1 | Amika | 4 | 1 | Teico | 8 | 0 | 1 | 0 | 9 | 0 |
| 0 | | | 0 | | | 0 | 0 | 0 | 6 | 0 |
| 0 | | | 0 | | | 0 | 0 | 0 | 6 | 0 |
| 0 | | | 0 | | | 0 | 0 | 0 | 7 | 0 |
| 0 | | | 0 | | | 0 | 1 | 0 | 6 | 0 |
| 0 | | | 0 | | | 0 | 0 | 0 | 6 | 0 |
| 0 | | | 0 | | | 0 | 0 | 0 | 30 | 0 |
| 0 | | | 0 | | | 0 | 0 | 0 | 17 | 0 |
| 0 | | | 0 | | | 0 | 0 | 0 | 8 | 0 |
| 0 | | | 0 | | | 0 | 0 | 0 | 10 | 0 |
| 0 | | | 0 | | | 0 | 0 | 0 | 6 | 0 |
| 0 | | | 0 | | | 0 | 0 | 0 | 5 | 0 |
| 1 | Amika | 3 | 1 | Teico | 5 | 0 | 0 | 0 | 6 | 0 |
| 0 | | | 0 | | | 0 | 0 | 0 | 6 | 0 |
| 1 | Amika | 3 | 1 | Teico | 6 | 0 | 0 | 0 | 2 | 0 |
| 0 | | | 1 | Teico | 6 | 0 | 0 | 0 | 6 | 0 |
| 1 | Amika | 5 | 1 | Teico | 5 | 0 | 0 | 0 | 7 | 0 |
| 0 | | | 1 | Teico | 5 | 0 | 0 | 0 | 7 | 0 |
| 0 | | | 0 | | | 0 | 0 | 0 | 2 | 0 |
| 0 | | | 0 | | | 0 | 0 | 0 | 4 | 0 |
| 0 | | | 0 | | | 0 | 0 | 0 | 3 | 0 |
| 0 | | | 0 | | | 0 | 0 | 0 | 4 | 0 |
| 0 | | | 1 | Teico | 6 | 0 | 0 | 0 | 15 | 0 |
| 0 | | | 0 | | | 0 | 0 | 0 | 5 | 0 |
| 0 | | | 0 | | | 0 | 0 | 0 | 3 | 0 |
| 0 | | | 0 | | | 0 | 0 | 0 | 2 | 0 |
| 0 | | | 0 | | | 0 | 0 | 0 | 0 | 1 |
| 0 | | | 0 | | | 0 | 0 | 2 | 2 | 0 |
| 0 | | | 0 | | | 0 | 0 | 0 | 0 | 1 |
| 0 | | | 0 | | | 0 | 0 | 0 | 0 | 1 |
| 0 | | | 0 | | | 0 | 0 | 0 | 0 | 1 |
| 0 | | | 0 | | | 0 | 0 | 0 | 0 | 1 |
| 0 | | | 1 | Teico | 3 | 0 | 0 | 0 | 0 | 1 |

| | | | | | | | | | | |
|---|---|---|---|---|---|---|---|---|---|---|
| 0 | | | 1 | Teico | 5 | 0 | 1 | 0 | 0 | 1 |
| 0 | | | 1 | Teico | 9 | 0 | 0 | 0 | 0 | 1 |
| 0 | | | 0 | | | 0 | 0 | 0 | 0 | 1 |
| 0 | | | 1 | Teico | 10 | 0 | 0 | 0 | 0 | 1 |
| 0 | | | 1 | Teico | 5 | 0 | 0 | 0 | 0 | 1 |
| 0 | | | 0 | | | 0 | 0 | 0 | 0 | 1 |
| 0 | | | 0 | | | 0 | 0 | 0 | 0 | 1 |
| 0 | | | 0 | | | 0 | 0 | 0 | 0 | 1 |
| 0 | | | 0 | | | 0 | 0 | 0 | 0 | 1 |
| 0 | | | 0 | | | 0 | 0 | 0 | 0 | 1 |
| 0 | | | 0 | | | 0 | 0 | 0 | 0 | 1 |
| 0 | | | 0 | | | 0 | 0 | 0 | 0 | 1 |
| 0 | | | 1 | Teico | 9 | 0 | 0 | 0 | 0 | 1 |
| 0 | | | 0 | | | 0 | 0 | 0 | 0 | 1 |
| 0 | | | 1 | Teico | 8 | 0 | 0 | 0 | 0 | 1 |
| 0 | | | 0 | | | 0 | 0 | 0 | 0 | 1 |
| 0 | | | 0 | | | 0 | 0 | 0 | 5 | 1 |
| 0 | | | 1 | Teico | 4 | 0 | 1 | 0 | 0 | 1 |
| 0 | | | 0 | | | 0 | 0 | 0 | 0 | 1 |
| 0 | | | 0 | | | 0 | 0 | 0 | 0 | 1 |
| 0 | | | 0 | | | 0 | 0 | 0 | 0 | 1 |
| 0 | | | 0 | | | 0 | 0 | 0 | 0 | 1 |
| 1 | Metro | 5 | 1 | Teico | 5 | 0 | 0 | 0 | 0 | 1 |
| 0 | | | 1 | Teico | 5 | 0 | 0 | 0 | 0 | 1 |
| 0 | | | 0 | | | 0 | 0 | 0 | 0 | 1 |
| 0 | | | 1 | Teico | 4 | 0 | 0 | 0 | 0 | 1 |
| 0 | | | 0 | | | 0 | 0 | 0 | 0 | 2 |
| 0 | | | 0 | | | 0 | 0 | 0 | 0 | 2 |
| 0 | | | 0 | | | 0 | 0 | 0 | 0 | 2 |
| 0 | | | 0 | | | 0 | 0 | 0 | 0 | 2 |
| 0 | | | 1 | Teico | 5 | 0 | 0 | 0 | 0 | 2 |
| 0 | | | 0 | | | 0 | 0 | 0 | 0 | 2 |
| 0 | | | 0 | | | 0 | 0 | 0 | 0 | 2 |
| 0 | | | 0 | | | 0 | 0 | 0 | 0 | 2 |
| 0 | | | 0 | | | 0 | 0 | 0 | 0 | 2 |
| 0 | | | 0 | | | 0 | 0 | 0 | 0 | 2 |
| 0 | | | 0 | | | 0 | 0 | 0 | 0 | 2 |
| 0 | | | 0 | | | 0 | 0 | 0 | 0 | 2 |
| 0 | | | 0 | | | 0 | 0 | 0 | 0 | 2 |
| 0 | | | 0 | | | 0 | 0 | 0 | 0 | 2 |

| | | | | | | | | | |
|---|---|---|---|---|---|---|---|---|---|
| 0 | | | 0 | | | 0 | 0 | 0 | 0 | 2 |
| 0 | | | 0 | | | 0 | 0 | 0 | 0 | 2 |
| 0 | | | 0 | | | 0 | 0 | 0 | 0 | 2 |
| 0 | | | 0 | | | 0 | 0 | 0 | 0 | 2 |
| 0 | | | 0 | | | 0 | 0 | 0 | 0 | 2 |
| 0 | | | 0 | | | 0 | 0 | 0 | 0 | 2 |
| 0 | | | 0 | | | 0 | 0 | 0 | 0 | 2 |
| 0 | | | 0 | | | 0 | 0 | 0 | 0 | 2 |
| 0 | | | 0 | | | 0 | 0 | 0 | 0 | 2 |
| 0 | | | 0 | | | 0 | 0 | 0 | 0 | 2 |
| 0 | | | 0 | | | 0 | 0 | 0 | 0 | 2 |
| 1 | Amika | 3 | 1 | Teico | 7 | 0 | 1 | 0 | 0 | 2 |
| 0 | | | 0 | | | 0 | 0 | 0 | 0 | 2 |
| 0 | | | 0 | | | 0 | 0 | 0 | 0 | 2 |
| 0 | | | 0 | | | 0 | 0 | 0 | 0 | 2 |
| 0 | | | 1 | Teico | 5 | 0 | 0 | 0 | 0 | 2 |
| 0 | | | 0 | | | 0 | 0 | 0 | 0 | 2 |
| 0 | | | 0 | | | 0 | 0 | 0 | 0 | 2 |
| 0 | | | 0 | | | 0 | 0 | 0 | 0 | 2 |
| 0 | | | 0 | | | 0 | 0 | 0 | 0 | 2 |
| 0 | | | 0 | | | 0 | 0 | 0 | 0 | 2 |
| 0 | | | 0 | | | 0 | 0 | 0 | 0 | 2 |
| 0 | | | 0 | | | 0 | 0 | 0 | 0 | 2 |
| 0 | | | 0 | | | 0 | 0 | 0 | 0 | 2 |
| 1 | Azitro | 3 | 1 | Meropenem | 7 | 0 | 0 | 4 | 0 | 2 |
| 0 | | | 0 | | | 0 | 0 | 0 | 0 | 2 |
| 0 | | | 0 | | | 0 | 0 | 0 | 0 | 2 |
| 0 | | | 0 | | | 0 | 0 | 0 | 0 | 2 |
| 0 | | | 0 | | | 0 | 0 | 0 | 0 | 2 |
| 0 | | | 0 | | | 0 | 0 | 0 | 0 | 2 |
| 0 | | | 0 | | | 0 | 0 | 0 | 0 | 2 |
| 0 | | | 0 | | | 0 | 0 | 0 | 0 | 2 |

| Mucositis | Mucositis2 | Other toxicity | her toxicity gra | BC transfusio | C trans numb | telet transfus | transfusion n | Readmission | Cause |
|---|---|---|---|---|---|---|---|---|---|
| 1 | 0 | 0 | | 0 | 0 | 1 | 2 | 0 | 0 |
| 0 | 0 | 0 | | 1 | 2 | 1 | 2 | 0 | 0 |
| 1 | 0 | 0 | | 0 | 0 | 1 | 1 | 0 | 0 |
| 0 | 0 | 0 | | 0 | 0 | 1 | 2 | 0 | 0 |
| 1 | 0 | 0 | | 0 | 0 | 1 | 1 | 0 | 0 |
| 0 | 0 | 0 | | 0 | 0 | 1 | 2 | 0 | 0 |
| 1 | 0 | 0 | | 0 | 0 | 1 | 1 | 0 | 0 |
| 1 | 0 | 0 | | 1 | 2 | 1 | 1 | 0 | 0 |
| 1 | 0 | 0 | | 0 | 0 | 1 | 1 | 0 | 0 |
| 1 | 0 | 0 | | 0 | 0 | 0 | 0 | 0 | 0 |
| 1 | 0 | 1 | 1 | 0 | 0 | 1 | 1 | 0 | 0 |
| 0 | 0 | 0 | | 0 | 0 | 0 | 0 | 0 | 0 |
| 1 | 0 | 1 | 1 | 1 | 6 | 1 | 15 | 1 | 1 |
| 1 | 0 | 3 | 1 | 1 | 3 | 1 | 5 | 0 | 0 |
| 2 | 1 | 3 | 1 | 1 | 2 | 1 | 2 | 0 | 0 |
| 0 | 0 | 0 | | 0 | 0 | 1 | 3 | 0 | 0 |
| 0 | 0 | 0 | | 0 | 0 | 1 | 1 | 0 | 0 |
| 1 | 0 | 0 | | 0 | 0 | 1 | 1 | 0 | 0 |
| 1 | 0 | 0 | | 0 | 0 | 1 | 2 | 1 | 1 |
| 0 | 0 | 3 | 1 | 0 | 0 | 1 | 2 | 0 | 0 |
| 0 | 0 | 0 | | 0 | 0 | 1 | 1 | 1 | 4 |
| 1 | 0 | 0 | | 0 | 0 | 1 | 2 | 0 | 0 |
| 0 | 0 | 3 | 2 | 0 | 0 | 1 | 2 | 1 | 1 |
| 0 | 0 | 3 | 1 | 0 | 0 | 1 | 2 | 0 | 0 |
| 0 | 0 | 0 | | 0 | 0 | 1 | 2 | 0 | 0 |
| 0 | 0 | 0 | | 0 | 0 | 1 | 1 | 0 | 0 |
| 2 | 1 | 3 | 1 | 0 | 0 | 1 | 1 | 0 | 0 |
| 0 | 0 | 3 | 1 | 0 | 0 | 1 | 2 | 0 | 0 |
| 0 | 0 | 3 | 1 | 0 | 0 | 1 | 4 | 0 | 0 |
| 1 | 0 | 0 | | 0 | 0 | 1 | 1 | 0 | 0 |
| 0 | 0 | 0 | | 0 | 0 | 1 | 2 | 0 | 0 |
| 0 | 0 | 0 | | 0 | 0 | 1 | 2 | 0 | 0 |
| 0 | 0 | 0 | | 0 | 0 | 1 | 1 | 0 | 0 |
| 0 | 0 | 0 | | 0 | 0 | 1 | 1 | 0 | 0 |
| 0 | 0 | 3 | 1 | 0 | 0 | 1 | 1 | 0 | 0 |
| 0 | 0 | 0 | | 0 | 0 | 1 | 1 | 0 | 0 |
| 0 | 0 | 0 | | 0 | 0 | 1 | 2 | 0 | 0 |
| 0 | 0 | 3 | 1 | 0 | 0 | 1 | 2 | 0 | 0 |
| 0 | 0 | 0 | | 0 | 0 | 1 | 3 | 0 | 0 |

| | | | | | | | | | |
|---|---|---|---|---|---|---|---|---|---|
| 0 | 0 | 3 | 1 | 1 | 1 | 1 | 3 | 0 | 0 |
| 1 | 0 | 3 | 1 | 0 | 0 | 1 | 2 | 0 | 0 |
| 1 | 0 | 3 | 1 | 0 | 0 | 1 | 2 | 0 | 0 |
| 0 | 0 | 0 | | 1 | 1 | 1 | 2 | 0 | 0 |
| 0 | 0 | 3 | 1 | 0 | 0 | 1 | 1 | 0 | 0 |
| 0 | 0 | 3 | 1 | 0 | 0 | 1 | 2 | 0 | 0 |
| 0 | 0 | 3 | 1 | 0 | 0 | 1 | 1 | 0 | 0 |
| 0 | 0 | 3 | 1 | 0 | 0 | 1 | 1 | 0 | 0 |
| 0 | 0 | 0 | | 0 | 0 | 1 | 1 | 0 | 0 |
| 0 | 0 | 0 | | 0 | 0 | 1 | 1 | 0 | 0 |
| 0 | 0 | 0 | | 0 | 0 | 1 | 3 | 0 | 0 |
| 0 | 0 | 0 | | 0 | 0 | 1 | 1 | 0 | 0 |
| 0 | 0 | 0 | | 0 | 0 | 1 | 2 | 0 | 0 |
| 0 | 0 | 3 | 1 | 0 | 0 | 1 | 3 | 0 | 0 |
| 0 | 0 | 0 | | 0 | 0 | 1 | 2 | 0 | 0 |
| 0 | 0 | 0 | | 0 | 0 | 1 | 1 | 0 | 0 |
| 0 | 0 | 3 | 1 | 0 | 0 | 1 | 1 | 0 | 0 |
| 0 | 0 | 0 | | 0 | 0 | 1 | 1 | 0 | 0 |
| 0 | 0 | 0 | | 1 | 1 | 1 | 2 | 0 | 0 |
| 0 | 0 | 3 | 1 | 0 | 0 | 1 | 1 | 0 | 0 |
| 0 | 0 | 0 | | 0 | 0 | 1 | 2 | 0 | 0 |
| 0 | 0 | 0 | | 0 | 0 | 1 | 2 | 0 | 0 |
| 0 | 0 | 3 | 1 | 0 | 0 | 1 | 2 | 1 | 3 |
| 0 | 0 | 0 | | 0 | 0 | 1 | 2 | 0 | 0 |
| 0 | 0 | 0 | | 0 | 0 | 1 | 1 | 0 | 0 |
| 0 | 0 | 0 | | 0 | 0 | 1 | 2 | 0 | 0 |
| 0 | 0 | 0 | | 0 | 0 | 1 | 1 | 0 | 0 |
| 0 | 0 | 0 | | 0 | 0 | 1 | 1 | 0 | 0 |
| 0 | 0 | 0 | | 0 | 0 | 1 | 1 | 0 | 0 |
| 1 | 0 | 3 | 1 | 0 | 0 | 1 | 1 | 0 | 0 |
| 0 | 0 | 0 | | 1 | 1 | 1 | 2 | 0 | 0 |
| 0 | 0 | 0 | | 0 | 0 | 1 | 1 | 0 | 0 |
| 0 | 0 | 3 | 2 | 0 | 0 | 1 | 1 | 0 | 0 |
| 0 | 0 | 4 | 1 | 0 | 0 | 1 | 2 | 0 | 0 |
| 0 | 0 | 0 | | 0 | 0 | 1 | 3 | 0 | 0 |
| 0 | 0 | 0 | | 0 | 0 | 0 | 0 | 0 | 0 |
| 0 | 0 | 0 | | 0 | 0 | 1 | 2 | 0 | 0 |
| 1 | 0 | 3 | 1 | 0 | 0 | 1 | 1 | 0 | 0 |
| 1 | 0 | 3 | 1 | 0 | 0 | 1 | 2 | 0 | 0 |
| 0 | 0 | 3 | 2 | 0 | 0 | 1 | 1 | 0 | 0 |

| | | | | | | | | | |
|---|---|---|---|---|---|---|---|---|---|
| 0 | 0 | 3 | 2 | 0 | 0 | 1 | 1 | 0 | 0 |
| 0 | 0 | 3 | 2 | 0 | 0 | 1 | 1 | 0 | 0 |
| 0 | 0 | 0 | | 0 | 0 | 1 | 1 | 0 | 0 |
| 0 | 0 | 3 | 1 | 0 | 0 | 0 | 0 | 0 | 0 |
| 0 | 0 | 0 | | 0 | 0 | 1 | 1 | 0 | 0 |
| 0 | 0 | 3 | 1 | 0 | 0 | 1 | 2 | 0 | 0 |
| 0 | 0 | 3 | 2 | 0 | 0 | 1 | 1 | 0 | 0 |
| 0 | 0 | 0 | | 0 | 0 | 1 | 1 | 0 | 0 |
| 0 | 0 | 0 | | 0 | 0 | 1 | 3 | 0 | 0 |
| 0 | 0 | 0 | | 0 | 0 | 1 | 1 | 0 | 0 |
| 0 | 0 | 0 | | 0 | 0 | 1 | 1 | 0 | 0 |
| 0 | 0 | 0 | | 0 | 0 | 1 | 2 | 0 | 0 |
| 0 | 0 | 0 | | 0 | 0 | 1 | 2 | 0 | 0 |
| 0 | 0 | 0 | | 0 | 0 | 1 | 1 | 0 | 0 |
| 0 | 0 | 0 | | 0 | 0 | 1 | 1 | 0 | 0 |
| 0 | 0 | 0 | | 0 | 0 | 1 | 5 | 0 | 0 |
| 0 | 0 | 4 | 3 | 0 | 0 | 1 | 1 | 0 | 0 |
| 0 | 0 | 0 | | 0 | 0 | 1 | 1 | 0 | 0 |
| 0 | 0 | 0 | | 0 | 0 | 1 | 1 | 0 | 0 |
| 1 | 0 | 3 | 1 | 0 | 0 | 1 | 1 | 0 | 0 |
| 0 | 0 | 3 | 1 | 0 | 0 | 1 | 1 | 0 | 0 |
| 0 | 0 | 0 | | 0 | 0 | 1 | 1 | 0 | 0 |
| 2 | 1 | 3 | 1 | 0 | 0 | 0 | 0 | 0 | 0 |
| 1 | 0 | 4 | 1 | 0 | 0 | 1 | 1 | 0 | 0 |
| 0 | 0 | 3 | 1 | 1 | 5 | 1 | 12 | 1 | 3 |
| 0 | 0 | 0 | | 0 | 0 | 1 | 1 | 0 | 0 |
| 0 | 0 | 0 | | 0 | 0 | 1 | 3 | 0 | 0 |
| 0 | 0 | 3 | 2 | 0 | 0 | 1 | 1 | 0 | 0 |
| 0 | 0 | 3 | 2 | 0 | 0 | 1 | 1 | 0 | 0 |
| 2 | 1 | 3 | 2 | 0 | 0 | 1 | 4 | 0 | 0 |
| 0 | 0 | 3 | 2 | 0 | 0 | 1 | 3 | 0 | 0 |
| 0 | 0 | 0 | | 0 | 0 | 1 | 1 | 0 | 0 |

| admission durati | ASCT athome days | IgH | IgL | ISS | Stage | Treatment preASC | Novel Drugs | Previous lines | Mobilization |
|---|---|---|---|---|---|---|---|---|---|
| 0 | 13 | 0 | 0 | 3 | 4 | 1 | 0Old | 2 | 1 |
| 0 | 14 | 0 | 0 | 1 | 3 | 3 | 1ND | 2 | 1 |
| 0 | 14 | 0 | 1 | 2 | 1 | 1 | 0Old | 1 | 1 |
| 0 | 11 | 2 | 1 | 1 | 3 | 1 | 0Old | 1 | 1 |
| 0 | 14 | 1 | 1 | 1 | 1 | 1 | 0Old | 1 | 1 |
| 0 | 11 | 0 | 0 | 2 | 1 | 1 | 0Old | 1 | 1 |
| 0 | 14 | 1 | 0 | 1 | 1 | 1 | 0Old | 1 | 1 |
| 0 | 14 | 0 | 1 | 1 | 1 | 1 | 0Old | 1 | 1 |
| 0 | 14 | 0 | 1 | 1 | 1 | 1 | 0Old | 1 | 1 |
| 0 | 14 | 2 | 1 | 1 | 1 | 1 | 0Old | 2 | 3 |
| 0 | 14 | 2 | 0 | 1 | 3 | 1 | 0Old | 1 | 1 |
| 0 | 13 | 0 | 0 | 1 | 3 | 2 | 1ND | 1 | 1 |
| 13 | 18 | 4 | 0 | 1 | 1 | 2 | 1ND | 1 | 1 |
| 0 | 24 | 2 | 0 | 1 | 1 | 1 | 0Old | 1 | 1 |
| 0 | 21 | 1 | 1 | 1 | 3 | 3 | 1ND | 1 | 1 |
| 0 | 17 | 2 | 0 | 1 | 1 | 2 | 1ND | 2 | 1 |
| 0 | 15 | 1 | 0 | 2 | 2 | 4 | 1ND | 1 | 1 |
| 0 | 14 | 2 | 0 | 1 | 3 | 1 | 0Old | 1 | 3 |
| 4 | 10 | 1 | 0 | 1 | 3 | 2 | 1ND | 4 | 1 |
| 0 | 14 | 0 | 0 | 1 | 3 | 1 | 0Old | 1 | 1 |
| 6 | 7 | 0 | 1 | 1 | 2 | 3 | 1ND | 1 | 1 |
| 0 | 17 | 0 | 0 | 1 | 3 | 2 | 1ND | 3 | 1 |
| 10 | 7 | 0 | 0 | 1 | 2 | 2 | 1ND | 1 | 1 |
| 0 | 15 | 1 | 1 | 1 | 3 | 2 | 1ND | 1 | 1 |
| 0 | 13 | 0 | 0 | 1 | 3 | 2 | 1ND | 1 | 1 |
| 0 | 13 | 2 | 1 | 1 | 2 | 2 | 1ND | 1 | 1 |
| 0 | 22 | 1 | 0 | 1 | 3 | 2 | 1ND | 1 | 1 |
| 0 | 15 | 0 | 1 | 2 | 3 | 2 | 1ND | 1 | 1 |
| 0 | 17 | 0 | 0 | 1 | 1 | 1 | 0Old | 1 | 1 |
| 0 | 14 | 0 | 0 | 1 | 3 | 2 | 1ND | 3 | 1 |
| 0 | 14 | 1 | 1 | 3 | 1 | 2 | 1ND | 1 | 1 |
| 0 | 15 | 0 | 1 | 1 | 3 | 2 | 1ND | 1 | 1 |
| 0 | 13 | 1 | 0 | 1 | 2 | 2 | 1ND | 2 | 1 |
| 0 | 18 | 0 | 1 | 1 | 3 | 2 | 1ND | 2 | 1 |
| 0 | 13 | 0 | 1 | 1 | 2 | 4 | 1ND | 1 | 1 |
| 0 | 14 | 1 | 1 | 1 | 2 | 4 | 1ND | 2 | 1 |
| 0 | 15 | 0 | 1 | 1 | 3 | 1 | 0Old | 2 | 1 |
| 0 | 17 | 1 | 0 | 3 | 1 | 4 | 1ND | 1 | 1 |
| 0 | 18 | 0 | 2 | 2 | 1 | 4 | 1ND | 1 | 3 |

| | | | | | | | | | |
|---|---|---|---|---|---|---|---|---|---|
| 0 | 27 | 0 | 1 | 2 | 3 | 4 | 1ND | 2 | 1 |
| 0 | 18 | 0 | 0 | 1 | 3 | 1 | 0Old | 2 | 3 |
| 0 | 15 | 0 | 1 | 1 | 1 | 2 | 1ND | 1 | 4 |
| 0 | 20 | 0 | 0 | 3 | 4 | 2 | 1ND | 1 | 1 |
| 0 | 14 | 0 | 0 | 1 | 3 | 2 | 1ND | 1 | 1 |
| 0 | 15 | 0 | 0 | 2 | 3 | 2 | 1ND | 1 | 1 |
| 0 | 15 | 0 | 0 | 1 | 3 | 2 | 1ND | 1 | 2 |
| 0 | 14 | 2 | 1 | 1 | 1 | 4 | 1ND | 1 | 1 |
| 0 | 15 | 1 | 0 | 1 | 3 | 2 | 1ND | 1 | 3 |
| 0 | 19 | 0 | 0 | 1 | 3 | 4 | 1ND | 1 | 1 |
| 0 | 13 | 2 | 1 | 1 | 2 | 2 | 1ND | 2 | 1 |
| 0 | 13 | 0 | 1 | 2 | 2 | 4 | 1ND | 1 | 1 |
| 0 | 16 | 1 | 0 | 1 | 4 | 4 | 1ND | 3 | 1 |
| 0 | 22 | 1 | 1 | 1 | 3 | 1 | 0Old | 3 | 2 |
| 2 | 16 | 0 | 1 | 1 | 3 | 4 | 1ND | 1 | 1 |
| 0 | 15 | 1 | 1 | 2 | 1 | 4 | 1ND | 1 | 1 |
| 0 | 13 | 2 | 0 | 1 | 1 | 4 | 1ND | 1 | 1 |
| 0 | 15 | 2 | 1 | 1 | 2 | 4 | 1ND | 1 | 1 |
| 0 | 14 | 2 | 0 | 1 | 2 | 2 | 1ND | 1 | 1 |
| 0 | 14 | 0 | 1 | 1 | 2 | 2 | 1ND | 1 | 1 |
| 0 | 16 | 1 | 0 | 2 | 1 | 4 | 1ND | 1 | 1 |
| 0 | 14 | 0 | 0 | 1 | 3 | 4 | 1ND | 1 | 1 |
| 3 | 15 | 0 | 0 | 1 | 3 | 4 | 1ND | 1 | 1 |
| 0 | 16 | 0 | 1 | 1 | 3 | 4 | 1ND | 1 | 1 |
| 0 | 16 | 1 | 1 | 2 | 2 | 4 | 1ND | 1 | 1 |
| 0 | 15 | 0 | 0 | 1 | 2 | 2 | 1ND | 1 | 1 |
| 0 | 17 | 0 | 0 | 1 | 1 | 4 | 1ND | 1 | 1 |
| 0 | 14 | 2 | 0 | 1 | 2 | 4 | 1ND | 1 | 1 |
| 0 | 16 | 2 | 0 | 1 | 1 | 4 | 1ND | 1 | 1 |
| 0 | 15 | 2 | 0 | 1 | 1 | 4 | 1ND | 1 | 1 |
| 0 | 18 | 2 | 1 | 3 | 1 | 2 | 1ND | 1 | 1 |
| 0 | 15 | 0 | 1 | 3 | 3 | 4 | 1ND | 1 | 1 |
| 0 | 16 | 0 | 0 | 2 | 2 | 4 | 1ND | 1 | 1 |
| 0 | 15 | 4 | 0 | 1 | 2 | 4 | 1ND | 3 | 2 |
| 0 | 17 | 1 | 0 | 3 | 2 | 4 | 1ND | 1 | 1 |
| 0 | 16 | 0 | 0 | 1 | 2 | 4 | 1ND | 1 | 1 |
| 0 | 15 | 1 | 0 | 1 | 1 | 4 | 1ND | 1 | 2 |
| 0 | 15 | 4 | 0 | 1 | 2 | 4 | 1ND | 3 | 2 |
| 0 | 14 | 1 | 1 | 2 | 2 | 4 | 1ND | 2 | 2 |
| 0 | 18 | 2 | 0 | 1 | 1 | 4 | 1ND | 4 | 2 |

| | | | | | | | | | |
|---|---|---|---|---|---|---|---|---|---|
| 0 | 14 | 2 | 0 | 2 | 1 | 4 | 1ND | 1 | 1 |
| 0 | 18 | 0 | 0 | 2 | 3 | 4 | 1ND | 1 | 2 |
| 0 | 14 | 1 | 0 | 2 | 1 | 4 | 1ND | 1 | 1 |
| 0 | 14 | 1 | 0 | 1 | 2 | 4 | 1ND | 1 | 1 |
| 0 | 17 | 1 | 0 | 3 | 2 | 4 | 1ND | 1 | 1 |
| 0 | 14 | 1 | 1 | 1 | 2 | 4 | 1ND | 1 | 1 |
| 0 | 16 | 1 | 1 | 1 | 2 | 4 | 1ND | 1 | 1 |
| 0 | 14 | 0 | 0 | 1 | 2 | 4 | 1ND | 1 | 1 |
| 0 | 18 | 1 | 1 | 1 | 2 | 4 | 1ND | 1 | 2 |
| 0 | 14 | 0 | 0 | 2 | 3 | 4 | 1ND | 1 | 2 |
| 0 | 17 | 1 | 0 | 2 | 2 | 4 | 1ND | 1 | 1 |
| 0 | 15 | 1 | 0 | 3 | 2 | 4 | 1ND | 1 | 1 |
| 0 | 16 | 2 | 1 | 2 | 2 | 4 | 1ND | 1 | 1 |
| 0 | 14 | 2 | 0 | 1 | 2 | 4 | 1ND | 1 | 1 |
| 0 | 14 | 0 | 1 | 2 | 3 | 4 | 1ND | 2 | 1 |
| 0 | 19 | 2 | 1 | 2 | 1 | 4 | 1ND | 1 | 2 |
| 0 | 16 | 0 | 0 | 2 | 2 | 4 | 1ND | 4 | 1 |
| 0 | 15 | 2 | 1 | 1 | 2 | 4 | 1ND | 1 | 1 |
| 0 | 14 | 0 | 0 | 1 | 3 | 4 | 1ND | 1 | 1 |
| 0 | 13 | 0 | 0 | 1 | 3 | 4 | 1ND | 1 | 1 |
| 0 | 14 | 0 | 0 | 3 | 2 | 4 | 1ND | 1 | 1 |
| 0 | 14 | 0 | 0 | 3 | 3 | 4 | 1ND | 1 | 1 |
| 0 | 14 | 1 | 1 | 2 | 1 | 4 | 1ND | 2 | 1 |
| 0 | 18 | 0 | 0 | 2 | 4 | 4 | 1ND | 3 | 1 |
| 12 | 13 | 2 | 0 | 3 | 2 | 4 | 1ND | 4 | 1 |
| 0 | 16 | 0 | 0 | 2 | 2 | 4 | 1ND | 1 | 1 |
| 0 | 21 | 2 | 0 | 2 | 1 | 1 | 0Old | 3 | 1 |
| 0 | 15 | 0 | 0 | 2 | 4 | 4 | 1ND | 6 | 2 |
| 0 | 17 | 0 | 0 | 3 | 3 | 4 | 1ND | 2 | 1 |
| 0 | 21 | 0 | 0 | 1 | 3 | 4 | 1ND | 1 | 1 |
| 0 | 15 | 2 | 1 | 2 | 2 | 4 | 1ND | 2 | 1 |
| 0 | 17 | 0 | 1 | 1 | 2 | 4 | 1ND | 1 | 4 |

| Sorror | Anti-emetic | Cryotherapy | Death | Death cause | ate Death/LastVis | Progression | Progression/La | graftment syndro |
|---|---|---|---|---|---|---|---|---|
| 0 | 0 | 0 | 1 | 2 | 08/06/2012 | 1 | 02/01/2002 | 1 |
| 2 | 0 | 0 | 0 | 0 | 04/09/2018 | 1 | 11/11/2002 | 0 |
| 2 | 0 | 0 | 0 | 0 | 11/08/2006 | 1 | 03/08/2005 | 1 |
| 0 | 0 | 0 | 0 | 0 | 27/10/2004 | 0 | 27/10/2004 | 1 |
| 0 | 0 | 0 | 0 | 0 | 04/10/2006 | 1 | 09/01/2003 | 0 |
| 0 | 0 | 0 | 1 | 2 | 09/08/2015 | 1 | 29/10/2003 | 0 |
| 0 | 0 | 0 | 1 | 2 | 06/02/2016 | 1 | 01/07/2015 | 1 |
| 0 | 0 | 0 | 1 | 2 | 03/06/2016 | 1 | 10/10/2009 | 0 |
| 2 | 0 | 1 | 1 | 2 | 14/01/2005 | 1 | 14/01/2005 | 0 |
| 2 | 0 | 1 | 1 | 2 | 17/06/2009 | 1 | 10/06/2006 | 0 |
| 0 | 0 | 1 | 0 | 0 | 20/04/2018 | 1 | 01/01/2007 | 1 |
| 0 | 0 | 1 | 0 | 0 | 04/07/2018 | 1 | 03/01/2009 | 0 |
| 0 | 0 | 1 | 1 | 2 | 01/08/2008 | 1 | 01/08/2007 | 1 |
| 0 | 0 | 1 | 1 | 2 | 06/01/2010 | 1 | 07/08/2008 | 1 |
| 3 | 0 | 1 | 0 | 0 | 02/10/2018 | 0 | 02/10/2018 | 1 |
| 0 | 0 | 1 | 0 | 0 | 08/05/2018 | 0 | 08/05/2018 | 0 |
| 4 | 0 | 1 | 1 | 2 | 20/12/2010 | 1 | 14/12/2009 | 0 |
| 0 | 0 | 1 | 1 | 2 | 31/01/2011 | 1 | 21/09/2010 | 1 |
| 3 | 0 | 1 | 1 | 2 | 06/02/2016 | 1 | 01/02/2012 | 1 |
| 0 | 0 | 1 | 0 | 0 | 19/03/2018 | 0 | 19/03/2018 | 1 |
| 2 | 0 | 1 | 0 | 0 | 23/07/2018 | 0 | 23/07/2018 | 1 |
| 0 | 0 | 1 | 0 | 0 | 20/06/2018 | 0 | 20/06/2018 | 0 |
| 0 | 0 | 1 | 0 | 0 | 20/04/2018 | 1 | 01/11/2018 | 1 |
| 0 | 0 | 1 | 0 | 0 | 25/09/2018 | 1 | 25/09/2018 | 1 |
| 2 | 1 | 1 | 0 | 0 | 31/10/2018 | 0 | 31/10/2018 | 1 |
| 0 | 1 | 1 | 0 | 0 | 10/07/2018 | 1 | 10/11/2013 | 0 |
| 0 | 1 | 1 | 0 | 0 | 10/10/2018 | 1 | 01/06/2013 | 0 |
| 0 | 1 | 1 | 0 | 0 | 12/08/2011 | 0 | 12/08/2011 | 1 |
| 1 | 1 | 1 | 0 | 0 | 31/10/2018 | 0 | 31/10/2018 | 1 |
| 0 | 1 | 1 | 0 | 0 | 21/09/2018 | 1 | 01/01/2016 | 0 |
| 3 | 1 | 1 | 0 | 0 | 23/10/2018 | 0 | 23/10/2018 | 1 |
| 0 | 1 | 1 | 1 | 2 | 26/04/2016 | 1 | 26/04/2016 | 1 |
| 0 | 1 | 1 | 1 | 2 | 30/05/2018 | 1 | 01/11/2013 | 0 |
| 0 | 1 | 1 | 0 | 0 | 27/07/2018 | 1 | 01/03/2013 | 0 |
| 1 | 1 | 1 | 0 | 0 | 15/02/2018 | 0 | 15/02/2018 | 0 |
| 0 | 1 | 1 | 0 | 0 | 19/09/2018 | 1 | 05/05/2013 | 1 |
| 3 | 1 | 1 | 1 | 2 | 22/02/2013 | 1 | 01/09/2012 | 0 |
| 0 | 1 | 1 | 1 | 2 | 12/12/2016 | 1 | 12/12/2016 | 1 |
| 2 | 1 | 1 | 0 | 0 | 25/10/2018 | 1 | 13/08/2014 | 0 |

| | | | | | | | | |
|---|---|---|---|---|---|---|---|---|
| 3 | 1 | 1 | 0 | 0 | 26/09/2018 | 1 | 01/06/2015 | 1 |
| 0 | 1 | 1 | 1 | 2 | 29/06/2013 | 1 | 13/12/2012 | 1 |
| 4 | 1 | 1 | 0 | 0 | 17/09/2018 | 1 | 17/09/2018 | 1 |
| 2 | 1 | 1 | 0 | 0 | 22/10/2018 | 1 | 15/01/2015 | 0 |
| 3 | 1 | 1 | 0 | 0 | 17/09/2018 | 0 | 17/09/2018 | 0 |
| 0 | 1 | 1 | 1 | 2 | 07/03/2015 | 1 | 01/09/2013 | 0 |
| 0 | 1 | 1 | 0 | 0 | 17/09/2018 | 0 | 17/09/2018 | 0 |
| 0 | 1 | 1 | 0 | 0 | 05/11/2018 | 0 | 05/11/2018 | 1 |
| 0 | 1 | 1 | 1 | 2 | 02/05/2014 | 1 | 01/12/2013 | 0 |
| 2 | 1 | 1 | 0 | 0 | 13/06/2013 | 0 | 13/06/2013 | 0 |
| 2 | 1 | 1 | 1 | 2 | 14/04/2014 | 1 | 14/01/2014 | 1 |
| 0 | 1 | 1 | 0 | 0 | 31/10/2018 | 0 | 31/10/2018 | 0 |
| 0 | 1 | 1 | 1 | 2 | 08/01/2014 | 1 | 01/10/2013 | 0 |
| 3 | 1 | 1 | 0 | 0 | 29/10/2018 | 0 | 29/10/2018 | 1 |
| 1 | 1 | 1 | 0 | 0 | 23/08/2018 | 1 | 01/10/2015 | 0 |
| 0 | 1 | 1 | 1 | 2 | 08/12/2017 | 1 | 27/03/2018 | 0 |
| 0 | 1 | 1 | 0 | 0 | 05/09/2018 | 1 | 08/02/2018 | 0 |
| 0 | 1 | 1 | 0 | 0 | 17/10/2018 | 1 | 02/02/2017 | 1 |
| 2 | 1 | 1 | 0 | 0 | 23/07/2018 | 0 | 23/07/2018 | 1 |
| 0 | 1 | 1 | 0 | 0 | 23/10/2018 | 1 | 05/09/2018 | 1 |
| 0 | 1 | 1 | 0 | 0 | 13/09/2018 | 1 | 13/09/2018 | 0 |
| 3 | 1 | 1 | 0 | 0 | 25/10/2018 | 1 | 01/11/2016 | 0 |
| 0 | 1 | 1 | 0 | 0 | 27/09/2018 | 0 | 27/09/2018 | 1 |
| 6 | 1 | 1 | 0 | 0 | 17/10/2018 | 0 | 17/10/2018 | 1 |
| 2 | 1 | 1 | 0 | 0 | 13/07/2018 | 0 | 13/07/2018 | 0 |
| 3 | 1 | 1 | 0 | 0 | 21/11/2014 | 0 | 21/11/2014 | 1 |
| 2 | 1 | 1 | 0 | 0 | 21/09/2018 | 0 | 21/09/2018 | 0 |
| 0 | 1 | 1 | 0 | 0 | 26/09/2018 | 0 | 26/09/2018 | 0 |
| 2 | 1 | 1 | 0 | 0 | 02/08/2018 | 0 | 02/08/2018 | 0 |
| 2 | 1 | 1 | 0 | 0 | 18/09/2018 | 0 | 18/09/2018 | 0 |
| 5 | 1 | 1 | 0 | 0 | 25/09/2018 | 1 | 10/10/2016 | 1 |
| 2 | 1 | 1 | 0 | 0 | 10/10/2018 | 0 | 10/10/2018 | 0 |
| 2 | 1 | 1 | 0 | 0 | 02/05/2016 | 0 | 02/05/2016 | 0 |
| 0 | 1 | 1 | 0 | 0 | 25/10/2018 | 0 | 25/10/2018 | 0 |
| 2 | 1 | 1 | 0 | 0 | 16/08/2018 | 0 | 16/08/2018 | 0 |
| 2 | 1 | 1 | 0 | 0 | 18/10/2018 | 0 | 18/10/2018 | 0 |
| 2 | 1 | 1 | 0 | 0 | 25/09/2018 | 0 | 25/09/2018 | 0 |
| 0 | 1 | 1 | 0 | 0 | 25/10/2018 | 0 | 25/10/2018 | 0 |
| 8 | 1 | 1 | 1 | 2 | 22/03/2018 | 1 | 20/11/2016 | 0 |
| 2 | 1 | 1 | 0 | 0 | 01/10/2018 | 0 | 01/10/2018 | 0 |

| | | | | | | | | |
|---|---|---|---|---|---|---|---|---|
| 2 | 1 | 1 | 0 | 0 | 19/09/2018 | 0 | 19/09/2018 | 1 |
| 2 | 1 | 1 | 0 | 0 | 03/11/2016 | 0 | 03/11/2016 | 0 |
| 3 | 1 | 1 | 0 | 0 | 01/10/2018 | 0 | 01/10/2018 | 1 |
| 1 | 1 | 1 | 0 | 0 | 24/10/2018 | 0 | 24/10/2018 | 0 |
| 2 | 1 | 1 | 0 | 0 | 03/09/2018 | 0 | 03/09/2018 | 0 |
| 3 | 1 | 1 | 0 | 0 | 23/10/2018 | 0 | 23/10/2018 | 0 |
| 2 | 1 | 1 | 0 | 0 | 04/10/2018 | 1 | 27/07/2018 | 1 |
| 0 | 1 | 1 | 0 | 0 | 03/10/2018 | 0 | 03/10/2018 | 0 |
| 3 | 1 | 1 | 0 | 0 | 08/05/2018 | 0 | 08/05/2018 | 0 |
| 0 | 1 | 1 | 0 | 0 | 26/09/2018 | 0 | 26/09/2018 | 0 |
| 0 | 1 | 1 | 0 | 0 | 15/05/2018 | 0 | 15/05/2018 | 0 |
| 3 | 1 | 1 | 0 | 0 | 03/09/2018 | 0 | 03/09/2018 | 1 |
| 2 | 1 | 1 | 0 | 0 | 16/10/2018 | 0 | 16/10/2018 | 0 |
| 3 | 1 | 1 | 0 | 0 | 27/09/2018 | 0 | 27/09/2018 | 1 |
| 2 | 1 | 1 | 0 | 0 | 17/10/2018 | 0 | 17/10/2018 | 0 |
| 2 | 1 | 1 | 0 | 0 | 03/10/2018 | 1 | 01/05/2018 | 1 |
| 3 | 1 | 1 | 0 | 0 | 10/10/2018 | 0 | 10/10/2018 | 0 |
| 2 | 1 | 1 | 0 | 0 | 03/09/2018 | 0 | 03/09/2018 | 0 |
| 0 | 1 | 1 | 0 | 0 | 26/04/2018 | 0 | 26/04/2018 | 0 |
| 2 | 1 | 1 | 0 | 0 | 20/10/2017 | 0 | 20/10/2017 | 0 |
| 2 | 1 | 1 | 0 | 0 | 10/07/2018 | 0 | 10/07/2018 | 0 |
| 3 | 1 | 1 | 0 | 0 | 06/09/2018 | 0 | 06/09/2018 | 0 |
| 0 | 1 | 1 | 0 | 0 | 29/08/2018 | 0 | 29/08/2018 | 0 |
| 0 | 1 | 1 | 0 | 0 | 17/09/2018 | 0 | 17/09/2018 | 0 |
| 4 | 1 | 1 | 0 | 0 | 03/10/2018 | 0 | 03/10/2018 | 1 |
| 2 | 1 | 1 | 0 | 0 | 27/09/2018 | 0 | 27/09/2018 | 0 |
| 3 | 1 | 1 | 0 | 0 | 09/10/2018 | 0 | 09/10/2018 | 1 |
| 0 | 1 | 1 | 0 | 0 | 01/10/2018 | 0 | 01/10/2018 | 0 |
| 0 | 1 | 1 | 0 | 0 | 08/11/2018 | 0 | 08/11/2018 | 0 |
| 3 | 1 | 1 | 0 | 0 | 05/10/2018 | 0 | 05/10/2018 | 0 |
| 0 | 1 | 1 | 0 | 0 | 31/10/2018 | 0 | 31/10/2018 | 1 |
| 2 | 1 | 1 | 0 | 0 | 21/09/2018 | 0 | 21/09/2018 | 0 |

| Figure1 Event | Figure1 Time day | Figure2A Event | Figure2A Time d | Figure2B Event | Figure2B Time d | Figure2C Event | Figure2C Time |
|---|---|---|---|---|---|---|---|
| 1 | 9 | 1 | 6 | 1 | 6 | 0 | 50 |
| 1 | 9 | 0 | 35 | 0 | 60 | 0 | 50 |
| 1 | 6 | 1 | 7 | 1 | 7 | 0 | 50 |
| 1 | 10 | 1 | 9 | 1 | 9 | 0 | 50 |
| 1 | 8 | 0 | 35 | 0 | 60 | 0 | 50 |
| 1 | 8 | 0 | 35 | 0 | 60 | 0 | 50 |
| 1 | 9 | 1 | 5 | 1 | 5 | 0 | 50 |
| 1 | 8 | 0 | 35 | 0 | 60 | 0 | 50 |
| 1 | 5 | 0 | 35 | 0 | 60 | 0 | 50 |
| 1 | 8 | 0 | 35 | 0 | 60 | 0 | 50 |
| 1 | 11 | 1 | 8 | 1 | 8 | 0 | 50 |
| 1 | 9 | 0 | 35 | 0 | 60 | 0 | 50 |
| 1 | 22 | 1 | 9 | 1 | 9 | 1 | 12 |
| 1 | 18 | 1 | 12 | 1 | 12 | 0 | 50 |
| 1 | 10 | 1 | 7 | 1 | 7 | 0 | 50 |
| 1 | 10 | 0 | 35 | 0 | 60 | 0 | 50 |
| 1 | 7 | 0 | 35 | 0 | 60 | 0 | 50 |
| 1 | 7 | 1 | 8 | 1 | 8 | 0 | 50 |
| 1 | 8 | 1 | 8 | 1 | 8 | 1 | 8 |
| 1 | 8 | 1 | 7 | 1 | 7 | 0 | 50 |
| 1 | 8 | 1 | 4 | 1 | 4 | 1 | 7 |
| 1 | 7 | 1 | 7 | 0 | 60 | 0 | 50 |
| 1 | 7 | 1 | 7 | 1 | 7 | 1 | 7 |
| 1 | 8 | 1 | 8 | 1 | 8 | 0 | 50 |
| 1 | 7 | 1 | 7 | 1 | 7 | 0 | 50 |
| 1 | 7 | 1 | 7 | 0 | 60 | 0 | 50 |
| 1 | 7 | 0 | 35 | 0 | 60 | 0 | 50 |
| 1 | 6 | 1 | 7 | 1 | 12 | 0 | 50 |
| 1 | 10 | 1 | 8 | 1 | 8 | 0 | 50 |
| 1 | 8 | 0 | 35 | 0 | 60 | 0 | 50 |
| 1 | 7 | 1 | 9 | 1 | 9 | 0 | 50 |
| 1 | 9 | 1 | 8 | 1 | 8 | 0 | 50 |
| 1 | 12 | 0 | 35 | 0 | 60 | 0 | 50 |
| 1 | 11 | 0 | 35 | 0 | 60 | 0 | 50 |
| 1 | 10 | 0 | 35 | 0 | 60 | 0 | 50 |
| 1 | 10 | 0 | 35 | 1 | 9 | 0 | 50 |
| 1 | 10 | 0 | 35 | 0 | 60 | 0 | 50 |
| 1 | 13 | 1 | 9 | 1 | 9 | 0 | 50 |
| 1 | 15 | 0 | 35 | 0 | 60 | 0 | 50 |

| | | | | | | | |
|---|---|---|---|---|---|---|---|
| 1 | 15 | 1 | 11 | 1 | 19 | 0 | 50 |
| 1 | 14 | 1 | 8 | 1 | 8 | 0 | 50 |
| 1 | 11 | 1 | 7 | 1 | 7 | 0 | 50 |
| 1 | 15 | 1 | 9 | 0 | 60 | 0 | 50 |
| 1 | 8 | 0 | 35 | 0 | 60 | 0 | 50 |
| 1 | 11 | 0 | 35 | 0 | 60 | 0 | 50 |
| 1 | 13 | 0 | 35 | 0 | 60 | 0 | 50 |
| 1 | 6 | 1 | 9 | 1 | 9 | 0 | 50 |
| 1 | 12 | 0 | 35 | 0 | 60 | 0 | 50 |
| 1 | 16 | 0 | 35 | 0 | 60 | 0 | 50 |
| 1 | 11 | 1 | 3 | 1 | 9 | 0 | 50 |
| 1 | 8 | 0 | 35 | 0 | 60 | 0 | 50 |
| 1 | 15 | 0 | 35 | 0 | 60 | 0 | 50 |
| 1 | 18 | 1 | 8 | 1 | 8 | 0 | 50 |
| 1 | 12 | 0 | 35 | 0 | 60 | 0 | 50 |
| 1 | 10 | 0 | 35 | 0 | 60 | 0 | 50 |
| 1 | 6 | 0 | 35 | 0 | 60 | 0 | 50 |
| 1 | 7 | 1 | 9 | 1 | 9 | 0 | 50 |
| 1 | 8 | 1 | 6 | 1 | 10 | 0 | 50 |
| 1 | 8 | 1 | 8 | 1 | 8 | 0 | 50 |
| 1 | 11 | 0 | 35 | 0 | 60 | 0 | 50 |
| 1 | 10 | 0 | 35 | 0 | 60 | 0 | 50 |
| 1 | 9 | 1 | 8 | 1 | 12 | 1 | 8 |
| 1 | 9 | 1 | 9 | 1 | 9 | 0 | 50 |
| 1 | 12 | 0 | 35 | 0 | 60 | 0 | 50 |
| 1 | 10 | 1 | 8 | 1 | 8 | 0 | 50 |
| 1 | 12 | 0 | 35 | 0 | 60 | 0 | 50 |
| 1 | 7 | 0 | 35 | 0 | 60 | 0 | 50 |
| 1 | 14 | 0 | 35 | 0 | 60 | 0 | 50 |
| 1 | 11 | 0 | 35 | 0 | 60 | 0 | 50 |
| 1 | 8 | 1 | 6 | 1 | 7 | 0 | 50 |
| 1 | 7 | 0 | 35 | 0 | 60 | 0 | 50 |
| 1 | 9 | 0 | 35 | 0 | 60 | 0 | 50 |
| 1 | 10 | 0 | 35 | 0 | 60 | 0 | 50 |
| 1 | 10 | 0 | 35 | 0 | 60 | 0 | 50 |
| 1 | 10 | 0 | 35 | 0 | 60 | 0 | 50 |
| 1 | 13 | 0 | 35 | 0 | 60 | 0 | 50 |
| 1 | 9 | 0 | 35 | 0 | 60 | 0 | 50 |
| 1 | 8 | 0 | 35 | 0 | 60 | 0 | 50 |
| 1 | 10 | 0 | 35 | 0 | 60 | 0 | 50 |

| | | | | | | | |
|---|---|---|---|---|---|---|---|
| 1 | 9 | 1 | 9 | 1 | 9 | 0 | 50 |
| 1 | 10 | 0 | 35 | 0 | 60 | 0 | 50 |
| 1 | 9 | 1 | 8 | 1 | 9 | 0 | 50 |
| 1 | 10 | 0 | 35 | 0 | 60 | 0 | 50 |
| 1 | 11 | 0 | 35 | 0 | 60 | 0 | 50 |
| 1 | 9 | 1 | 7 | 0 | 60 | 0 | 50 |
| 1 | 7 | 1 | 6 | 1 | 9 | 0 | 50 |
| 1 | 8 | 0 | 35 | 0 | 60 | 0 | 50 |
| 1 | 13 | 0 | 35 | 0 | 60 | 0 | 50 |
| 1 | 11 | 0 | 35 | 0 | 60 | 0 | 50 |
| 1 | 12 | 0 | 35 | 0 | 60 | 0 | 50 |
| 1 | 10 | 1 | 6 | 1 | 11 | 0 | 50 |
| 1 | 11 | 0 | 35 | 0 | 60 | 0 | 50 |
| 1 | 9 | 1 | 9 | 1 | 9 | 0 | 50 |
| 1 | 11 | 0 | 35 | 0 | 60 | 0 | 50 |
| 1 | 9 | 1 | 9 | 1 | 11 | 0 | 50 |
| 1 | 9 | 0 | 35 | 0 | 60 | 0 | 50 |
| 1 | 11 | 0 | 35 | 0 | 60 | 0 | 50 |
| 1 | 8 | 0 | 35 | 0 | 60 | 0 | 50 |
| 1 | 7 | 0 | 35 | 0 | 60 | 0 | 50 |
| 1 | 8 | 0 | 35 | 0 | 60 | 0 | 50 |
| 1 | 15 | 0 | 35 | 0 | 60 | 0 | 50 |
| 1 | 10 | 0 | 35 | 0 | 60 | 0 | 50 |
| 1 | 11 | 0 | 35 | 0 | 60 | 0 | 50 |
| 1 | 16 | 1 | 8 | 1 | 8 | 1 | 13 |
| 1 | 12 | 0 | 35 | 0 | 60 | 0 | 50 |
| 1 | 8 | 1 | 5 | 1 | 5 | 0 | 50 |
| 1 | 11 | 0 | 35 | 0 | 60 | 0 | 50 |
| 1 | 8 | 0 | 35 | 0 | 60 | 0 | 50 |
| 1 | **21** | 0 | 35 | 0 | 60 | 0 | 50 |
| 1 | 11 | 1 | 8 | 1 | 8 | 0 | 50 |
| 1 | 12 | 0 | 35 | 0 | 60 | 0 | 50 |

| Figure3PFSA Event | Figure3PFSA Time mc | Figure3PFSB Ev | Figure3PFSB Tir | Figure3OSA Eve | Figure3OSA Tim | Figure3OSB Eve | Figure3OSB Tim |
|---|---|---|---|---|---|---|---|
| 1 | 10,30 | 1 | 10,30 | 1 | 137,30 | 1 | 137,30 |
| 1 | 5,97 | 1 | 5,97 | 0 | 198,50 | 0 | 198,50 |
| 1 | 38,77 | 1 | 38,77 | 0 | 51,20 | 0 | 51,20 |
| 0 | 27,47 | 0 | 27,47 | 0 | 27,47 | 0 | 27,47 |
| 1 | 6,60 | 1 | 6,60 | 0 | 52,07 | 0 | 52,07 |
| 1 | 8,93 | 1 | 8,93 | 1 | 152,33 | 1 | 152,33 |
| 1 | 146,80 | 1 | 146,80 | 1 | 154,13 | 1 | 154,13 |
| 1 | 73,07 | 1 | 73,07 | 1 | 154,00 | 1 | 154,00 |
| 1 | 10,87 | 1 | 10,87 | 1 | 10,87 | 1 | 10,87 |
| 1 | 16,03 | 1 | 16,03 | 1 | 52,80 | 1 | 52,80 |
| 1 | 22,17 | 1 | 22,17 | 0 | 159,73 | 0 | 159,73 |
| 1 | 32,80 | 1 | 32,80 | 0 | 148,43 | 0 | 148,43 |
| 1 | 14,53 | 1 | 14,53 | 1 | 26,73 | 1 | 26,73 |
| 1 | 22,93 | 1 | 22,93 | 1 | 40,17 | 1 | 40,17 |
| 0 | 139,57 | 0 | 139,57 | 0 | 139,57 | 0 | 139,57 |
| 0 | 128,60 | 0 | 128,60 | 0 | 128,60 | 0 | 128,60 |
| 1 | 25,43 | 1 | 25,43 | 1 | 37,80 | 1 | 37,80 |
| 1 | 34,43 | 1 | 34,43 | 1 | 38,83 | 1 | 38,83 |
| 1 | 41,23 | 1 | 41,23 | 1 | 90,10 | 1 | 90,10 |
| 0 | 109,87 | 0 | 109,87 | 0 | 109,87 | 0 | 109,87 |
| 0 | 104,93 | 0 | 104,93 | 0 | 104,93 | 0 | 104,93 |
| 0 | 100,40 | 0 | 100,40 | 0 | 100,40 | 0 | 100,40 |
| 1 | 101,47 | 1 | 101,47 | 0 | 94,97 | 0 | 94,97 |
| 1 | 98,17 | 1 | 98,17 | 0 | 98,17 | 0 | 98,17 |
| 0 | 93,73 | 0 | 93,73 | 0 | 93,73 | 0 | 93,73 |
| 1 | 32,37 | 1 | 32,37 | 0 | 89,13 | 0 | 89,13 |
| 1 | 26,30 | 1 | 26,30 | 0 | 91,53 | 0 | 91,53 |
| 0 | 2,93 | 0 | 2,93 | 0 | 2,93 | 0 | 2,93 |
| 0 | 90,23 | 0 | 90,23 | 0 | 90,23 | 0 | 90,23 |
| 1 | 54,83 | 1 | 54,83 | 0 | 87,97 | 0 | 87,97 |
| 0 | 87,63 | 0 | 87,63 | 0 | 87,63 | 0 | 87,63 |
| 1 | 56,50 | 1 | 56,50 | 1 | 56,50 | 1 | 56,50 |
| 1 | 24,30 | 1 | 24,30 | 1 | 80,00 | 1 | 80,00 |
| 1 | 15,07 | 1 | 15,07 | 0 | 80,87 | 0 | 80,87 |
| 0 | 72,07 | 0 | 72,07 | 0 | 72,07 | 0 | 72,07 |
| 1 | 13,27 | 1 | 13,27 | 0 | 78,70 | 0 | 78,70 |
| 1 | 4,60 | 1 | 4,60 | 1 | 10,40 | 1 | 10,40 |
| 1 | 55,07 | 1 | 55,07 | 1 | 55,07 | 1 | 55,07 |
| 1 | 25,27 | 1 | 25,27 | 0 | 76,40 | 0 | 76,40 |

| | | | | | | | |
|---|---|---|---|---|---|---|---|
| 1 | 34,50 | 1 | 34,50 | 0 | 74,93 | 0 | 74,93 |
| 1 | 3,03 | 1 | 3,03 | 1 | 9,63 | 1 | 9,63 |
| 1 | 71,17 | 1 | 71,17 | 0 | 71,17 | 0 | 71,17 |
| 1 | 25,73 | 1 | 25,73 | 0 | 71,60 | 0 | 71,60 |
| 0 | 69,87 | 0 | 69,87 | 0 | 69,87 | 0 | 69,87 |
| 1 | 8,13 | 1 | 8,13 | 1 | 26,53 | 1 | 26,53 |
| 0 | 69,07 | 0 | 69,07 | 0 | 69,07 | 0 | 69,07 |
| 0 | 69,40 | 0 | 69,40 | 0 | 69,40 | 0 | 69,40 |
| 1 | 8,60 | 1 | 8,60 | 1 | 13,67 | 1 | 13,67 |
| 0 | 2,40 | 0 | 2,40 | 0 | 2,40 | 0 | 2,40 |
| 1 | 6,93 | 1 | 6,93 | 1 | 9,93 | 1 | 9,93 |
| 0 | 64,57 | 0 | 64,57 | 0 | 64,57 | 0 | 64,57 |
| 1 | 1,87 | 1 | 1,87 | 1 | 5,17 | 1 | 5,17 |
| 0 | 61,13 | 0 | 61,13 | 0 | 61,13 | 0 | 61,13 |
| 1 | 22,97 | 1 | 22,97 | 0 | 58,20 | 0 | 58,20 |
| 1 | 53,13 | 1 | 53,13 | 1 | 49,50 | 1 | 49,50 |
| 1 | 51,07 | 1 | 51,07 | 0 | 58,03 | 0 | 58,03 |
| 1 | 38,37 | 1 | 38,37 | 0 | 59,10 | 0 | 59,10 |
| 0 | 53,07 | 0 | 53,07 | 0 | 53,07 | 0 | 53,07 |
| 1 | 54,10 | 1 | 54,10 | 0 | 55,70 | 0 | 55,70 |
| 1 | 52,83 | 1 | 52,83 | 0 | 52,83 | 0 | 52,83 |
| 1 | 28,47 | 1 | 28,47 | 0 | 52,57 | 0 | 52,57 |
| 0 | 48,97 | 0 | 48,97 | 0 | 48,97 | 0 | 48,97 |
| 0 | 48,83 | 0 | 48,83 | 0 | 48,83 | 0 | 48,83 |
| 0 | 45,50 | 0 | 45,50 | 0 | 45,50 | 0 | 45,50 |
| 0 | 0,83 | 0 | 0,83 | 0 | 0,83 | 0 | 0,83 |
| 0 | 44,47 | 0 | 44,47 | 0 | 44,47 | 0 | 44,47 |
| 0 | 43,70 | 0 | 43,70 | 0 | 43,70 | 0 | 43,70 |
| 0 | 40,90 | 0 | 40,90 | 0 | 40,90 | 0 | 40,90 |
| 0 | 41,80 | 0 | 41,80 | 0 | 41,80 | 0 | 41,80 |
| 1 | 16,00 | 1 | 16,00 | 0 | 39,83 | 0 | 39,83 |
| 0 | 37,63 | 0 | 37,63 | 0 | 37,63 | 0 | 37,63 |
| 0 | 1,63 | 0 | 1,63 | 0 | 1,63 | 0 | 1,63 |
| 0 | 30,43 | 0 | 30,43 | 0 | 30,43 | 0 | 30,43 |
| 0 | 26,47 | 0 | 26,47 | 0 | 26,47 | 0 | 26,47 |
| 0 | 27,87 | 0 | 27,87 | 0 | 27,87 | 0 | 27,87 |
| 0 | 26,87 | 0 | 26,87 | 0 | 26,87 | 0 | 26,87 |
| 0 | 26,67 | 0 | 26,67 | 0 | 26,67 | 0 | 26,67 |
| 1 | 3,20 | 1 | 3,20 | 1 | 19,43 | 1 | 19,43 |
| 0 | 25,33 | 0 | 25,33 | 0 | 25,33 | 0 | 25,33 |

| | | | | | | | |
|---|---|---|---|---|---|---|---|
| 0 | 24,47 | 0 | 24,47 | 0 | 24,47 | 0 | 24,47 |
| 0 | 0,90 | 0 | 0,90 | 0 | 0,90 | 0 | 0,90 |
| 0 | 23,57 | 0 | 23,57 | 0 | 23,57 | 0 | 23,57 |
| 0 | 24,20 | 0 | 24,20 | 0 | 24,20 | 0 | 24,20 |
| 0 | 21,67 | 0 | 21,67 | 0 | 21,67 | 0 | 21,67 |
| 0 | 22,90 | 0 | 22,90 | 0 | 22,90 | 0 | 22,90 |
| 1 | 19,73 | 1 | 19,73 | 0 | 22,03 | 0 | 22,03 |
| 0 | 20,73 | 0 | 20,73 | 0 | 20,73 | 0 | 20,73 |
| 0 | 15,20 | 0 | 15,20 | 0 | 15,20 | 0 | 15,20 |
| 0 | 19,77 | 0 | 19,77 | 0 | 19,77 | 0 | 19,77 |
| 0 | 14,60 | 0 | 14,60 | 0 | 14,60 | 0 | 14,60 |
| 0 | 18,10 | 0 | 18,10 | 0 | 18,10 | 0 | 18,10 |
| 0 | 19,40 | 0 | 19,40 | 0 | 19,40 | 0 | 19,40 |
| 0 | 18,20 | 0 | 18,20 | 0 | 18,20 | 0 | 18,20 |
| 0 | 18,83 | 0 | 18,83 | 0 | 18,83 | 0 | 18,83 |
| 1 | 13,03 | 1 | 13,03 | 0 | 18,20 | 0 | 18,20 |
| 0 | 17,53 | 0 | 17,53 | 0 | 17,53 | 0 | 17,53 |
| 0 | 16,10 | 0 | 16,10 | 0 | 16,10 | 0 | 16,10 |
| 0 | 9,57 | 0 | 9,57 | 0 | 9,57 | 0 | 9,57 |
| 0 | 3,07 | 0 | 3,07 | 0 | 3,07 | 0 | 3,07 |
| 0 | 11,23 | 0 | 11,23 | 0 | 11,23 | 0 | 11,23 |
| 0 | 13,03 | 0 | 13,03 | 0 | 13,03 | 0 | 13,03 |
| 0 | 11,13 | 0 | 11,13 | 0 | 11,13 | 0 | 11,13 |
| 0 | 9,33 | 0 | 9,33 | 0 | 9,33 | 0 | 9,33 |
| 0 | 7,20 | 0 | 7,20 | 0 | 7,20 | 0 | 7,20 |
| 0 | 5,87 | 0 | 5,87 | 0 | 5,87 | 0 | 5,87 |
| 0 | 5,27 | 0 | 5,27 | 0 | 5,27 | 0 | 5,27 |
| 0 | 3,97 | 0 | 3,97 | 0 | 3,97 | 0 | 3,97 |
| 0 | 4,40 | 0 | 4,40 | 0 | 4,40 | 0 | 4,40 |
| 0 | 2,47 | 0 | 2,47 | 0 | 2,47 | 0 | 2,47 |
| 0 | 2,83 | 0 | 2,83 | 0 | 2,83 | 0 | 2,83 |
| 0 | 1,30 | 0 | 1,30 | 0 | 1,30 | 0 | 1,30 |

e
